# Enhanced hepatic respiratory capacity and altered lipid metabolism support metabolic homeostasis during short-term hypoxic stress

**DOI:** 10.1186/s12915-021-01192-0

**Published:** 2021-12-15

**Authors:** Katie A. O’Brien, Ben D. McNally, Alice P. Sowton, Antonio Murgia, James Armitage, Luke W. Thomas, Fynn N. Krause, Lucas A. Maddalena, Ian Francis, Stefan Kavanagh, Dominic P. Williams, Margaret Ashcroft, Julian L. Griffin, Jonathan J. Lyon, Andrew J. Murray

**Affiliations:** 1 Department of Physiology, Development and Neuroscience, University of Cambridge, Downing Street, Cambridge, CB2 3EG UK; 2 Department of Biochemistry and Cambridge Systems Biology Centre, University of Cambridge, Sanger Building Tennis Court Road, Cambridge, CB2 1GA UK; 3 Global Investigative Safety, GlaxoSmithKline R&D, Park Road, Ware, Hertfordshire, SG12 0DP UK; 4 Department of Medicine, University of Cambridge, Cambridge Biomedical Campus, Hills Road, Cambridge, CB2 0QQ UK; 5 Ultrastructure and Cellular Bioimaging, GlaxoSmithKline R&D, Park Road, Ware, Hertfordshire, SG12 0DP UK; 6 Oncology Safety Sciences, Clinical Pharmacology & Safety Sciences, R&D, AstraZeneca, CB2 OAA, Cambridge, UK; 7 Functional and Mechanistic Safety, Clinical Pharmacology & Safety Sciences, R&D, AstraZeneca, CB2 OAA, Cambridge, UK; 8 Section of Biomolecular Medicine, Department of Digestion, Metabolism and Reproduction, Imperial College London, South Kensington Campus, London, SW7 2AZ UK

**Keywords:** Hypoxia, Hepatic mitochondria, Mitochondrial respiratory chain, Mitochondrial supercomplexes, De novo lipogenesis

## Abstract

**Background:**

Tissue hypoxia is a key feature of several endemic hepatic diseases, including alcoholic and non-alcoholic fatty liver disease, and organ failure. Hypoxia imposes a severe metabolic challenge on the liver, potentially disrupting its capacity to carry out essential functions including fuel storage and the integration of lipid metabolism at the whole-body level. Mitochondrial respiratory function is understood to be critical in mediating the hepatic hypoxic response, yet the time-dependent nature of this response and the role of the respiratory chain in this remain unclear.

**Results:**

Here, we report that hepatic respiratory capacity is enhanced following short-term exposure to hypoxia (2 days, 10% O_2_) and is associated with increased abundance of the respiratory chain supercomplex III_2_+IV and increased cardiolipin levels. Suppression of this enhanced respiratory capacity, achieved via mild inhibition of mitochondrial complex III, disrupted metabolic homeostasis. Hypoxic exposure for 2 days led to accumulation of plasma and hepatic long chain acyl-carnitines. This was observed alongside depletion of hepatic triacylglycerol species with total chain lengths of 39-53 carbons, containing palmitic, palmitoleic, stearic, and oleic acids, which are associated with de novo lipogenesis. The changes to hepatic respiratory capacity and lipid metabolism following 2 days hypoxic exposure were transient, becoming resolved after 14 days in line with systemic acclimation to hypoxia and elevated circulating haemoglobin concentrations.

**Conclusions:**

The liver maintains metabolic homeostasis in response to shorter term hypoxic exposure through transient enhancement of respiratory chain capacity and alterations to lipid metabolism. These findings may have implications in understanding and treating hepatic pathologies associated with hypoxia.

**Supplementary Information:**

The online version contains supplementary material available at 10.1186/s12915-021-01192-0.

## Background

Tissue hypoxia can arise as a result of disrupted convective oxygen (O_2_) delivery and imposes an energetic challenge on the tissues [1]. The liver plays a key role in integrating fuel storage and systemic metabolism, in particular lipid metabolism and requires significant O_2_ to perform this role. This results in steep O_2_ gradients across liver lobules and a high susceptibility to hypoxia [2]. Hepatic hypoxia is a key feature of a number of medical conditions and has been implicated in the development of both alcoholic and non-alcoholic fatty liver disease [2].

It has been suggested that alterations in respiratory function play a key role in linking tissue hypoxia, oxidative stress and lipid metabolism in the liver, with the role of the mitochondria having been identified as a specific knowledge gap in current understanding of pathological hepatic hypoxia [3]. The key molecular mediators of the cellular hypoxia response are the hypoxia inducible factor (HIF) family of transcription factors and associated regulatory proteins [4, 5]. Reactive oxygen species production by mitochondrial complex I (CI) and III (CIII) can mediate activation of HIF1α and HIF2α, an effect demonstrated within minutes of hypoxic exposure across a range of cell types [6–9]. Mitochondrial oxidative capacity is rapidly targeted in hypoxia, with Na^+^ entry via the mitochondrial Na^+^/Ca^2+^ exchanger decreasing the mobility of free ubiquinone, and in doing so impairing electron transfer to CIII [10]. The electron transfer system (ETS), and particularly signalling via CIII, is therefore integral to the metabolic response to hypoxic exposure.

HIF activation is associated with alterations in metabolism and mitochondrial O_2_ consumption [11] in both cells [12, 13] and tissues including heart [14] and skeletal muscle [15, 16]. In the heart, HIF activation has been associated with the suppression of fatty acid oxidation mediated via downregulation of peroxisome proliferator-activated receptor α (PPARα) [14]. In skeletal muscle, constitutive HIF activation has been linked to increased reliance on glycolytic ATP production [15, 16] and suppression of mitochondrial respiratory capacity [16].

In the liver, the activation of HIF1α and HIF2α are associated with contrasting effects. HIF2α is purported to stimulate lipogenesis and inhibit fatty acid oxidation [17], whereas it has been suggested that HIF1α prevents excess lipid accumulation in fatty liver by suppressing the sterol response element binding protein (SREBP)-1c-dependent lipogenic pathway [18]. Through the use of HIF1α-null mice, HIF1α activity has been linked to suppression of PPARα in liver and consequential downregulation of its transcriptional targets, including carnitine palmitoyltransferase 1 (CPT1) [18]. However, this contrasts with reports of increased hepatic PPARα and CPT1 expression in rats following 3 days of hypoxic exposure [19]. These apparent contradictions may reflect time-dependent aspects of the HIF response. Indeed, it has been posited that HIF1α underpins the response to more acute (< 24 h) exposure whilst HIF2α coordinates longer-term changes in response to more sustained hypoxia [20]. Elucidating the time-dependent nature of the hepatic metabolic response to hypoxia is therefore an important area of investigation.

Here, we investigated the metabolic response of rat liver to short-term (2 days) and prolonged (14 days) exposure to inhalation hypoxia (10% O_2_). The role of the mitochondrial ETS in this response, and so the importance of altered respiratory capacity, was probed through the mild inhibition of CIII [21]. To assess the consequences for hepatic and systemic metabolic regulation, metabolomic profiles were examined in liver and plasma, with a particular focus on alterations in lipid metabolism.

We demonstrate that maintenance of hepatic metabolic homeostasis during short-term hypoxic exposure is dependent on enhanced respiratory capacity, a response associated with mitochondrial respiratory supercomplex formation. Notably, this effect was transient, with respiratory capacity returning to normoxic levels after 14 days exposure. Lipid homeostasis was substantially altered after 2 days of hypoxic exposure, and this was partially dependent on alterations in respiratory capacity.

## Results

### Hepatic mitochondrial response to shorter-term and more sustained hypoxic exposure and the role of the electron transfer system

**Fig. 1:**
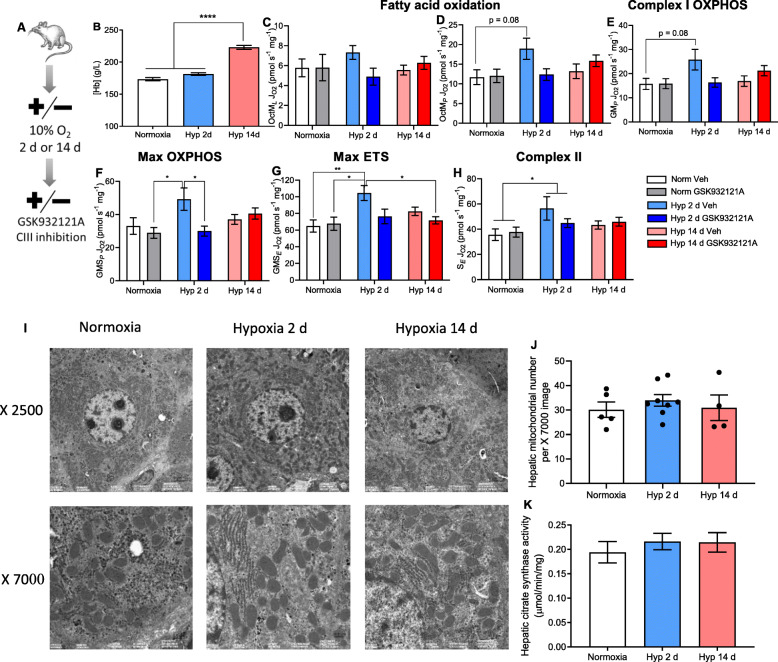
Shorter-term hypoxic exposure enhances hepatic mitochondrial respiratory capacity. **A** Experimental design. Female Crl:CD(SD) rats (220–300 g) were exposed to normoxia or hypoxia (10% O_2_) for either 2 days (2 d) or 14 days (14 d), after which an electron transfer system complex III (CIII) inhibitor (GSK932121A, 25 mg kg^−1^, *i.p.*) or vehicle (Veh) was administered. **B** Haemoglobin concentration ([Hb]) (g/L) obtained from the tail vein upon termination. Data set includes both vehicle and GSK932121A-treated groups, *n* = 18 normoxic, *n* = 16 for all other groups. **C**–**H** Respiration of liver homogenates from Veh and GSK932121A-treated rats in the following states: Octanoyl carnitine and malate-supported (β-oxidation) respiration, without ADP (OctM_*L*_) (**C**) and with ADP (OctM_*P*_) (**D**) to assess fatty acid oxidation. Stimulation of complex I-supported respiration with the addition of glutamate (GM_*P*_) (**E**). Stimulation of maximal OXPHOS following the addition of succinate (GMS_*P*_) (**F**) and maximal ETS capacity with the addition of the uncoupler FCCP (GMS_*E*_) (**G**). Finally, inhibition of complex I-supported respiration through the addition of rotenone, restricting electron flux through complex II in the presence of the electron transfer system uncoupler FCCP (S_*E*_) (**H**). Respiration rates are corrected to wet mass of liver tissue added to the chamber. *n* = 9 Veh normoxic rats, *n* = 8 Veh 2 d hypoxic, Veh and GSK932121A-treated 14 d hypoxic, *n* = 7 normoxic and 2 d hypoxic GSK932121A-treated rats. **I** Transmission electron microscopy of hepatic tissue at × 2500 and × 7000 magnifications. **J** Number of hepatic mitochondria per tissue area (μm^2^) using transmission electron microscopy. Data in Veh and GSK932121A-treated rats is combined, *n* = 5 normoxic, *n* = 8 hypoxic 2 d, *n* = 4 hypoxic 14 d. **K** Hepatic citrate synthase activity normalised to protein concentration. Data in Veh and GSK932121A-treated rats is combined, *n* = 17 normoxic, *n* = 16 hypoxic 2 d and 14 d. Data are presented as mean ± SEM, *p < 0.05, ** *p* ≤ 0.01, *** *p* ≤ 0.001.

Female Crl:CD(SD) rats (220–300 g, *n* = 8–10/group) were exposed to normoxia or hypoxia (10% O_2_) for either 2 days or 14 days. The degree and duration of hypoxic exposure were based upon prior work demonstrating time-dependent effects of 10% O_2_ exposure on respiratory capacity in rat heart and skeletal muscle [22]. Following hypoxic exposure, a mitochondrial CIII inhibitor (GSK932121A, 25 mg kg^−1^) or vehicle (Veh) was administered *i.p.* (Fig. 1A, see Methods). GSK932121A binds to the Qi site of CIII [23] and inhibits CIII catalytic activity in rodents [21] Prior work has demonstrated its toxic effects upon mitochondrial function in female Crl:CD(SD) rats when administered at a dose of 50 mg kg^−1^ *i.p* [21]. The dose used here is below that shown to invoke overt hepatotoxicity, with the aim being to exert mild CIII inhibition.

Sustained exposure to hypoxia induced an expected systemic response, including 27% higher blood haemoglobin concentrations ([Hb]) after 14 days compared with that of normoxic rats (*p* < 0.0001) (Fig. 1B). No rise in [Hb] was observed after 2 days of hypoxia, indicating that systemic acclimation to hypoxia had not occurred at this timepoint. Following GSK932121A or Veh administration, animals were euthanised after 1.5–3.5 h, based on the severity of clinical signs (ventilation rate, piloerection, hunched posture, orbital tightening and subdued behaviour [24]). Following 2 days of hypoxia, GSK932121A administration resulted in increased clinical signs compared with those seen in Veh-treated rats (*p* = 0.0002), a response that was greater than that seen in rats administered with GSK932121A after 14 days of hypoxia (*p* = 0.03) (Additional File 1: Figure S1A). At termination, levels of GSK932121A present in both liver and plasma did not differ between normoxic or hypoxic groups, suggesting metabolism of the drug was not impacted by hypoxic exposure (Additional File 1: Figure S1B,C). Following 2 days of hypoxia, GSK932121A administration resulted in 36% higher levels of liver hydroxyproline compared with that seen in Veh-treated hypoxic rats (*p* = 0.0003) (Additional File 1: Figure S1D), suggestive of the onset of hepatic fibrogenesis [25, 26]. There was, however, no increase in hepatic hydroxyproline when GSK932121A was administered to rats following 14 days of hypoxia. Liver fibrosis associated with mitochondrial dysfunction has been shown to coincide with raised circulating lactate [27–29]; however, no rise in plasma lactate was observed here following hypoxic exposure or GSK932121A administration, either alone or in combination (Additional File 1: Figure S1E). Administration of GSK932121A at 50 mg kg^−1^ has been associated with hepatic glycogen depletion [21], yet no change in hepatic glycogen storage was observed (Additional File 1: Figure S1F). Together, this suggests that whilst 2 days of hypoxic exposure in combination with GSK932121A administration invoked hepatic stress, the dose used did not result in overt hepatotoxicity.

To probe the response of hepatic mitochondria to hypoxia, we measured respiratory capacity in liver homogenates using high-resolution respirometry and a substrate-uncoupler-inhibitor titration (see Methods). Hepatic leak state respiration (supported by malate and octanoyl-carnitine, OctM_*L*_) was not altered by hypoxic exposure of either duration (Fig. 1C). Following 2 days of hypoxia, however, both hepatic fatty acid oxidation capacity (supported by malate and octanoyl-carnitine in the presence of ADP, OctM_*P*_, Fig. 1D) and respiration through the N-pathway via complex I (supported by malate and glutamate in the presence of ADP, GM_*P,*_ Fig. 1E) showed non-significant trends towards higher rates of respiration than those in normoxic rats (*p* = 0.08). Maximal oxidative phosphorylation (OXPHOS) capacity (supported by malate, glutamate, succinate and ADP, GMS_*P*_) (Fig. 1F) and ETS capacity (supported by malate, glutamate and succinate, ADP and the uncoupler FCCP, GMS_*E*_) (Fig. 1G) were both 58% higher in the livers of rats following 2 days of hypoxic exposure compared with normoxic rats (*p* < 0.05). This rise in hepatic respiratory capacity observed in 2 day hypoxia-exposed rats was sensitive to CIII inhibition, with GSK932121A-treatment eliminating the increased maximal OXPHOS capacity and ETS capacity compared with normoxic rats. Respiration through the S-pathway via complex II (CII), supported by succinate in the presence of rotenone (S_*E*_), was 40% higher in rats following 2 days of hypoxia compared with normoxic rats (*p* = 0.03) (Fig. 1H). Unlike the alterations in maximal OXPHOS and ETS capacities with shorter-term hypoxia, this increase in succinate-supported respiration was not sensitive to mild CIII inhibition with GSK932121A, suggesting that CIII was not saturated in this respiratory state. No alterations to hepatic mitochondrial respiratory capacity were sustained following 14 days of hypoxia, with respiration rates at this timepoint no different to those in the livers of normoxic rats.

To understand whether the enhanced hepatic respiratory capacity following 2 days of hypoxic exposure resulted from changes in liver mitochondrial content, the mitochondrial network was visualised using electron microscopy (Fig. 1I). No changes in mitochondrial number per area were observed in response to either 2 days or 14 days of hypoxic exposure (Fig. 1J). We also assessed citrate synthase activity, a putative marker of mitochondrial density [30] (Fig. 1K) and again found no change following hypoxic exposure at either timepoint. This suggests that the enhanced hepatic respiratory capacity observed in response to shorter-term hypoxia was not due to increased liver mitochondrial content.

At the onset of hypoxic exposure, ad libitum food intake decreased in rats by 55% during day 1 and by 43% during day 2, compared with pre-exposure intake (Additional File 1: Figure S2A). This corresponded to a 9.2% drop in body weight, which was recovered over subsequent days to 0.4% above baseline level by day 14 (Additional File 1: Figure S2B). To discern the effects of decreased food intake upon metabolic function over this 2 day period, female Crl:CD(SD) rats (220–300 g) were single-housed in normoxia for 2 days with food availability either matched to that consumed by hypoxic rats or freely available as controls (*n* = 6/group). In comparison to ad libitum-fed controls, pair-feeding rats to match the food intake of 2 day hypoxic animals induced a 27% rise in fatty acid oxidation-supported OXPHOS (*p* = 0.05) indicative of a fasted state. This was alongside a 23% rise in maximal ETS (*p* = 0.04). However, there was no alteration in maximal OXPHOS capacity (Additional File 1: Figure S2C), indicating that the rise in maximal OXPHOS capacity seen in hypoxic rats did not occur as a result of lower calorie intake.

### Respiratory complexes and supercomplexes following short-term hypoxia

**Fig. 2:**
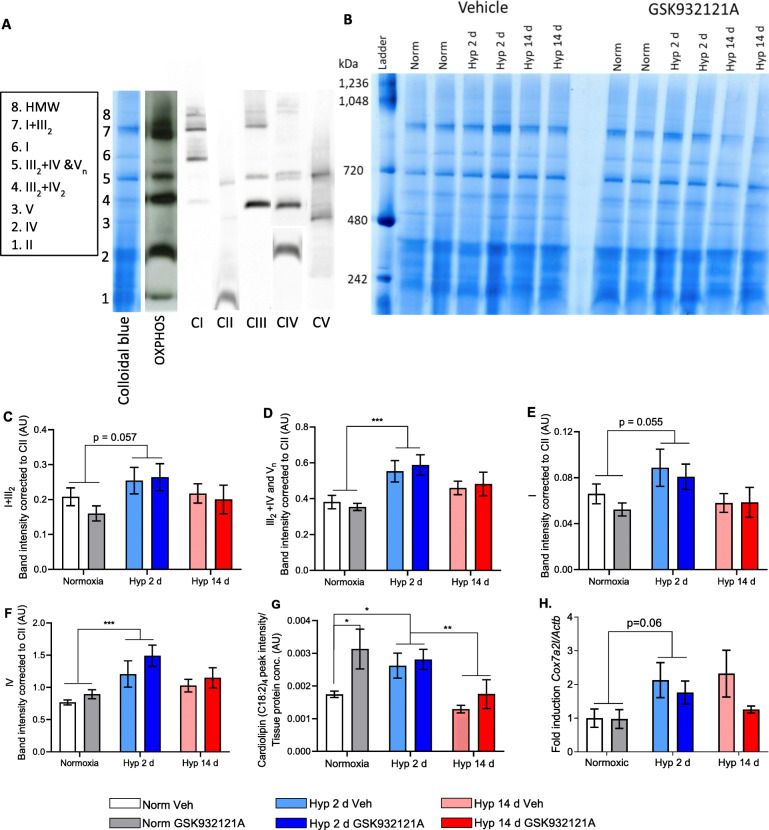
Short-term hypoxia is associated with hepatic mitochondrial supercomplex formation. **A** Immunoblotting of blue native polyacrylamide gel electrophoresis (BN-PAGE) gel using antibodies for each singular respiratory complex and the OXPHOS antibody cocktail to confirm identity of bands presented in the gel stained with colloidal blue. To avoid over-saturation of the bottom band, the Complex IV immunoblot is presented as two separate exposures. **B** Colloidal blue staining of a representative BN-PAGE gel, from which band density was quantified. **C**–**F**. Band intensity for the following mitochondrial complex and supercomplex stoichiometric combinations: I+III_2_ (C), III_2_+IV comigrated with V_n_ (**D**), alongside mitochondrial complexes I (**E**) and IV (**F**). Where the number of associated complexes is unknown, *n* is used. All bands are corrected to complex II levels. *n* = 10 Veh normoxic, *n* = 7 GSK932121A-treated 2 d and 14 d hypoxic, *n* = 8 for remaining groups. **G** Peak intensity of cardiolipin, measured by liquid chromatography-mass spectrometry (LC-MS), corrected to internal standard and protein concentration. *n* = 9 Veh normoxic, *n* = 7 GSK932121A-treated normoxic, *n* = 6 GSK932121A-treated 14 d hypoxia, *n* = 8 for remaining groups. **H** Expression levels of *Cox7a2l* measured by qPCR, presented as fold induction corrected to *Actb. n* = 7 GSK932121A-treated 14 d hypoxia, *n* = 8 for all remaining groups. Data are presented as mean ± SEM, *p < 0.05, ** *p* ≤ 0.01, *** *p* ≤ 0.001

In order to probe further mechanisms that might underpin the hepatic mitochondrial response to hypoxia, we sought to investigate the formation of mitochondrial supercomplexes and the expression of representative subunits of the ETS complexes. Hepatic respiratory supercomplexes were assessed through blue native polyacrylamide gel electrophoresis (BN-PAGE) on mitochondrial extracts solubilised using digitonin [31]. Band identification was achieved through immunoblotting (Fig. 2A and Additional File 1: Figure S3A) and comparison to recent characterisation of supercomplex structure [31, 32]. Analysis was performed using gels stained with Colloidal Blue (Fig. 2B).

Following 2 days of hypoxic exposure, the abundance of supercomplexes of the stoichiometric combinations I+III_2_ and III_2_+IV co-migrated with complex V (CV) were increased by 28% (*p* = 0.057) and 35% (*p* = 0.0002) respectively across Veh and GSK932121A-treated groups (Fig. 2C, D). Compared with normoxia, complex I (CI) monomer abundance showed a non-significant trend towards increasing, whilst complex IV (CIV) monomer abundance was 39% higher (*p* = 0.0005) following 2 d hypoxia (Fig. 2E, F). In response to pair-feeding, no significant change in supercomplex levels or monomer abundances were observed, although a non-significant trend towards increased abundance of CIV monomer of 18% (*p* = 0.052) was seen (Additional File 1: Figure S3B,C).

To assess whether band density changes were due to increased protein expression, the levels of representative subunits of complexes I–V were examined using immunoblotting. No statistical differences were seen in protein expression of any representative subunit following hypoxic exposure (Additional File 1: Figure S4A). This suggests the increase in band density observed for III_2_+IV co-migrated with CV was due to III_2_+IV formation rather than increased complex V expression.

Supercomplex assembly requires the phospholipid cardiolipin [33], synthesised on the inner mitochondrial membrane [34, 35]. The association of cardiolipin with the inner mitochondrial membrane is dependent on double bond composition, with the C18:2–C18:2 configuration being the principal species found in rat liver [36]. Following 2 days of hypoxic exposure, we found cardiolipin (C18:2)_4_ levels were 38% higher compared with those in Veh-treated normoxic rats and 44% higher in comparison with Veh-treated 14 day hypoxic rats (*p* < 0.05) (Fig. 2G). A structural component of supercomplexes that bridges complexes III and IV is COX7A2L/SCAF1 [37]. The expression of *Cox7a2l* examined by RT-qPCR revealed a trend towards increased expression following 2 days of hypoxia (*p* = 0.06), across both Veh and GSK932121A treated animals (Fig. 2H).

The assembly and stability of large supercomplexes is dependent on mitochondrial cristae shape [38] regulated through oligomerisation of the inner membrane optic atrophy 1 (OPA1) [39]. We therefore investigated factors known to be associated with OPA1 stability, including hypoxia-induced gene domain protein-1a (HIGD1A) [40], the mitochondrial solute carrier (SLC25A1) [39], and mitochondrial-localised protein stomatin-like protein 2 (STOML2) [41]. Hepatic expression of *Higd1a* increased by 62% following 2 days of hypoxia (*p* = 0.002), and this remained 55% higher following 14 days of hypoxia (*p* = 0.005) (Additional File 1: Figure S4B). In contrast, expression of *Slc25a11* and *Stoml2* did not change in response to hypoxic exposure (Additional File 1: Figure S4C,D).

Together, this implies that the enhanced hepatic mitochondrial respiratory capacity in response to 2 days of hypoxia exposure occurs via increased respiratory supercomplex formation and complex IV monomer abundance, accompanied by elevated levels of hepatic cardiolipin, *Cox7a2l* and *Higd1a*. Whilst mild inhibition of CIII suppressed respiratory function at 2 days of hypoxia, our results indicate that this inhibitor did not impact supercomplex formation.

### The mitochondrial response to short-term hypoxia is critical for hepatic metabolic homeostasis

**Fig. 3:**
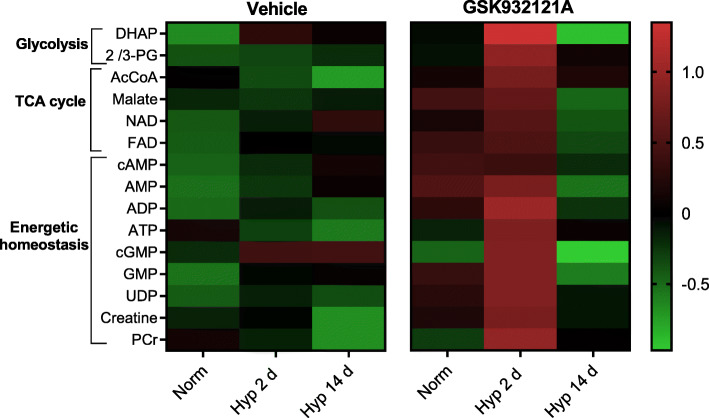
Maintenance of hepatic energetic homeostasis during short-term hypoxic stress is dependent on enhanced hepatic respiratory capacity. Targeted metabolomics performed using LC-MS, including presentation of dihydroxyacetone phosphate (DHAP), 2/3-phosphoglycerate (PG), acetyl CoA (AcCoA) and phosphocreatine (PCr). Data obtained from the peak area ratio, corrected to internal standards and protein concentration. For heatmap presentation, data was normalised using autoscaling and generalised logarithm transformation. *n* = 9 Veh normoxic, *n* = 8 all remaining groups. The 2 d hypoxic exposure with GSK932121A administration was defined as statistically significant across all metabolites presented through use of a two-way ANOVA plus false discovery rate correction (two-stage linear step-up procedure of Benjamini, Krieger, and Yekutieli, *Q* = 5%, threshold *p* value < 0.032), followed by a post-hoc Tukey’s test (*p* < 0.05)

To investigate the importance of the hepatic mitochondrial response to hypoxia for metabolic homeostasis, we employed targeted metabolomics of snap-frozen liver tissue to assess metabolites critical to glycolysis, tricarboxylic acid (TCA) cycle function, and energy metabolism, including adenosine, guanosine and uridine phosphates alongside creatine and phosphocreatine. Remarkably, levels of these metabolites were largely unaltered in rat liver following 2 days of hypoxic exposure, highlighting a robust hepatic response to maintain metabolic homeostasis in the face of hypoxia (Fig. 3).

HIF pathway activation in hypoxia is known to increase expression of glycolytic enzymes [15, 16, 42], whilst viability of cultured hepatocytes in acute hypoxia is dependent on glycolysis [43]. Surprisingly, hepatic expression of the glycolytic gene transcripts *Hk1*, *Pfk*, or *Ldha* were unaltered at 2 days or 14 days of hypoxic exposure (Additional File 1: Figure S5A-C), whilst expression of *Pgk1*, encoding phosphoglycerate kinase, was decreased by 55% following 2 days of hypoxia and by 49% after 14 days compared to normoxia (*p* > 0.05) (Additional File 1: Figure S5D). Along with the findings that glycolytic intermediates (Fig. 3) and glycogen storage (Additional File 1: Figure S1F) were unaltered at 2 d hypoxia, this suggests that the hepatic response to short-term hypoxic stress does not depend upon increased glycolytic capacity or flux.

Inhibition of CIII revealed the critical role of the hepatic mitochondrial response in maintaining energetic homeostasis, since GSK932121A administration to rats following 2 days of hypoxia (but not normoxic rats, nor rats following 14 days of hypoxia) resulted in widespread disruption of metabolic homeostasis. This was demonstrated through increased abundance of metabolites related to glycolysis, suggesting that inhibition of the enhanced respiratory capacity increases glycolytic flux. Moreover, the abundance of TCA cycle intermediates and metabolites related to energetic homeostasis were also altered following GSK932121A-treatment after 2 days of hypoxia (*p* < 0.05) (Fig. 3). This indicates that the enhanced hepatic respiratory capacity seen in response to short-term hypoxic stress is essential for the maintenance of metabolic homeostasis.

### Hepatic and systemic lipid metabolism is altered following 2 days of hypoxia

**Fig. 4:**
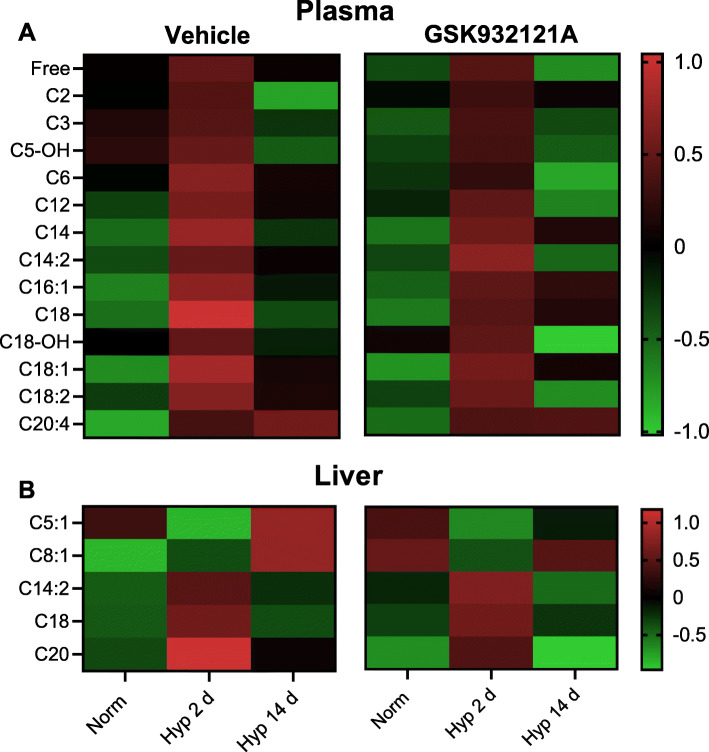
Plasma and liver acyl-carnitine profiles are altered by short-term hypoxia. **A** Plasma acyl-carnitines and free carnitine, *n* = 10 Veh normoxic, *n* = 8 all remaining groups. **B** Liver acyl-carnitines and free carnitine, *n* = 9 Veh normoxic, *n* = 8 all remaining groups. Intermediates in both panels were assessed by targeted metabolomics using liquid chromatography-mass spectrometry. Data presented is peak intensity obtained from the peak area ratio, corrected to internal standards and protein concentration and normalised using autoscaling and generalised logarithm transformation. The 2 d hypoxic exposure was defined as statistically significant across all metabolites presented through use of a two-way ANOVA plus false discovery rate correction (two-stage linear step-up procedure of Benjamini, Krieger and Yekutieli, *Q* = 5%, threshold *p* value < 0.032), followed by a post-hoc Tukey’s test (*p* < 0.05)

Next, we sought to investigate the effect of hypoxic exposure and the hepatic mitochondrial response to hypoxia on lipid metabolism, beginning with examination of fatty acid derived acyl-carnitines. Total plasma acyl-carnitines, including those with carbon chain lengths 2-20 (C2-20) and free/-L carnitine, were 33% higher following 2 days of hypoxia in comparison with the plasma of normoxic rats (*p* = 0.007). Notably, this was the case in both Veh-treated rats and those administered GSK932121A (Fig. 4A). Following 2 days of hypoxia, hepatic levels of short chain acyl-carnitines (C5,C8), were 78% lower (*p* = 0.003), whilst medium and long chain acyl-carnitines (C14-C20) were 47% higher than those in livers of normoxic animals (*p* = 0.006) (Fig. 4B). Again, this was unaffected by GSK932121A administration, suggesting that this occurs independently of the enhanced respiratory capacity seen at this timepoint.

**Fig. 5:**
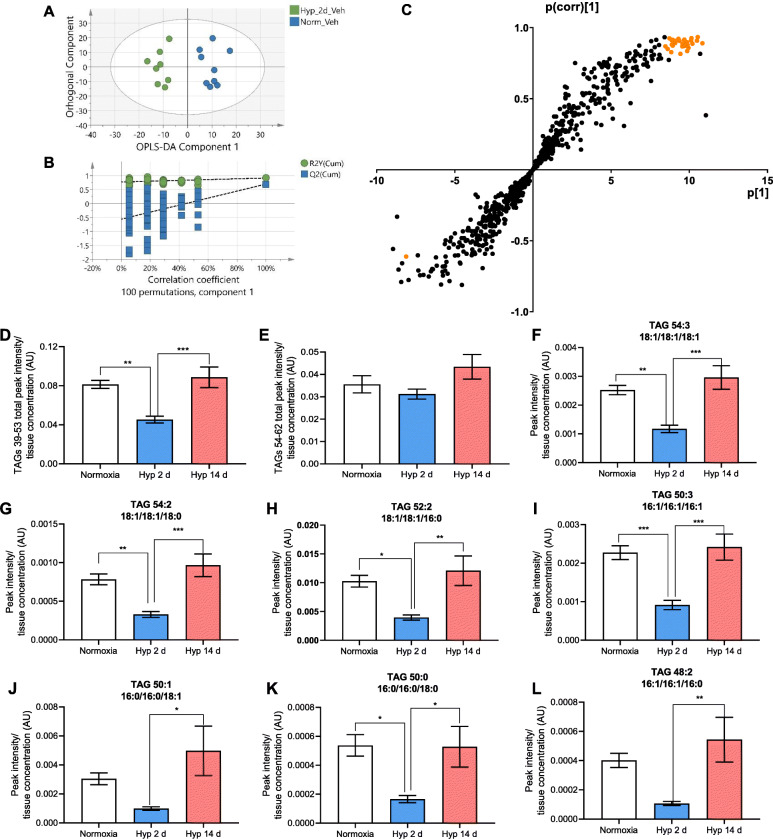
Hepatic triacylglycerol (TAG) composition is altered by short-term hypoxia with suppression of TAGs associated with de novo lipogenesis. **A** Orthogonal partial least squares—discriminant analysis (OPLS-DA) of hepatic lipidomic profiles of Veh-treated normoxic (green) and 2 d hypoxic (red) treated rats, with each dot representing the lipidomic profile of one animal. Data was obtained from the peak area ratio of LC-MS analysis, corrected to internal standards and protein concentration and normalised using Pareto Scaling and generalised logarithm transformation. **B** Permutation validation of the plot in **A**. **C** S-plot corresponding to the OPLS-DA model in **A**. Triacylglycerol species with discriminatory values, defined as those lying 2 standard deviations from the mean, are highlighted in orange. **D** Total peak intensities of TAGs with chain lengths 39-53. **E** Total peak intensities of TAGs with chain lengths 54-62. **F**–**L** TAGs with the greatest peak intensities alongside corresponding fatty acid composition derived from fragmentation analysis where 16:0 denotes palmitic acid, 16:1 palmitoleic acid, 18:1 oleic acid, 18:0 stearic acid. Data presented in **C**–**L** are Veh-treated animals in normoxia, after 2 d hypoxia and 14 d hypoxia. Peak intensities are corrected to internal standard and protein concentration. Bar graphs are presented as mean ± SEM, * *p* < 0.05, ** *p* ≤ 0.01, *** *p* ≤ 0.001. *n* = 9 Veh normoxic, *n* = 8 all remaining groups.

To further examine alterations in hepatic lipid metabolism following short-term hypoxic exposure, the hepatic lipidome was analysed in an open profile manner to compare Veh-treated normoxic and 2 day hypoxic rats. An orthogonal partial least squares-discriminant analysis (OPLS-DA) model readily separated these groups (*R*^2^*X*(cum) = 40.9%; Q^2^(cum) = 69.7%), Fig. 5A), and the model passed cross-validation by random permutation (Fig. 5B, *y*-axis intercepts *R*^2^ = 0.0, 0.76; *Q*^2^ = 0.0, − 0.45) and by CV-ANOVA (*p* = 0.004)). An S-plot was constructed and those lipid species lying 2 standard deviations from the mean were defined as driving the model separation (Fig. 5C). From this, triacylglycerols (TAGs) emerged as key discriminants. Subsequent analysis therefore focused upon TAGs and revealed that following 2 days of hypoxia, TAGs with carbon chain lengths 39-53 were 44% lower in abundance than they were in the livers of normoxic rats (*p* = 0.002) and 48% lower than in the livers of 14 day hypoxic rats (*p* = 0.0005) (Fig. 5D). This contrasts with hepatic TAGs of longer chain lengths 54-62, which were unchanged following hypoxic exposure (Fig. 5E). Fragmentation analysis was performed on the TAGs with the highest peak intensities to reveal their fatty acid compositions. This included palmitic (16:0), palmitoleic (16:1), stearic (18:0), and oleic (18:1) acids (Fig. 5F–L). TAGs with these specific fatty acid compositions have been directly associated with hepatic de novo lipogenesis (DNL) [44].

The fall in DNL-associated TAGs in response to 2 days of hypoxic exposure was driven by hypoxia rather than the associated fall in food consumption, as in normoxic rats pair-fed to match those exposed to hypoxia there was a 41% increase (*p* = 0.07) in TAGs of 39-53 chain length and a 70% increase (*p* = 0.007) in TAGs of 54-62 chain lengths (Additional File 1: Figure S6A,B).

To probe the effects of mild CIII inhibition on lipid homeostasis in short-term hypoxia, the lipid profiles of Veh and GSK932121A-treated animals following 2 days of hypoxia were compared. An OPLS-DA model readily separated these groups (*R*^2^*X*(cum) = 50.6%, *Q*^2^(cum) = 69.3%), (Additional File 1: Figure S7A), and the model passed cross-validation by random permutation (Additional File 1: Figure S7B), *y*-axis intercepts *R*^2^ = 0.0, 0.907 *Q*^2^ = 0.0, − 0.339) and by CV-ANOVA (*p* = 0.049). Key discriminants from this model, identified using the same S plot method as outlined above, were TAGs with 37-47 and 60-65 carbon chain lengths (Additional File 1: Figure S7C). This suggests that the enhanced respiratory capacity in response to short-term hypoxia influences the hepatic lipid profile, in particular the composition of the TAG pool. Mild CIII inhibition with GSK932121A did not, however, affect the fall in DNL-associated TAGs after 2 days of hypoxia, as GSK932121A-treated animals displayed a 46% decrease in TAGs of chain lengths 39-53 in comparison with normoxic animals (*p* = 0.01) (Additional File 1: Figure S7D). Moreover, GSK932121A administration induced a fall in abundance of these TAGs after 14 d hypoxia by 14% (*p* = 0.04). As with the Veh-treated animals, no change was observed in TAGs of 54-62 chain lengths with GSK932121A treatment (Additional File 1: Figure S7E).

Lipid abundance was consistently higher in the livers of normoxic, GSK932121A-treated rats compared with normoxic, Veh-treated rats. This was apparent in TAG levels (Fig. 5D, E and Additional File 1: Figure S7D,E), cardiolipin (Fig. 2G) and in total lipid abundance (Additional File 1: Figure S7F), the latter being 36% higher in normoxic, GSK932121A-treateded animals compared to Veh (*p* < 0.01). No comparable response to GSK932121A treatment was seen in either hypoxic group. This suggests that hepatic lipid homeostasis is disrupted in response to mild CIII inhibition, with the rise in total liver lipid intensity implying enhanced lipogenesis. However, this response was absent in the livers of hypoxic rats, perhaps suggesting that it is dependent upon an ample O_2_ supply.

## Discussion

Hypoxia is a key feature of common hepatic pathologies, with the time-dependent nature of the hepatic metabolic response to hypoxia being critical to our understanding of the aetiology of these conditions. Here, we report that hepatic respiratory capacity is enhanced following acute hypoxic exposure. This response was sensitive to mild CIII inhibition, which suppressed this rise in respiratory capacity and disrupted metabolic homeostasis. Hepatic mitochondrial content was unaltered by hypoxia, but instead, an increased abundance of mitochondrial supercomplexes, complex IV monomer and associated factors may underlie this enhanced respiratory capacity. Short-term hypoxia resulted in the accumulation of hepatic and plasma acyl-carnitines and other alterations to the hepatic lipid profile, including depletion of TAGs associated with DNL. The rise in respiratory capacity, increased sensitivity to CIII inhibition, and alterations to TAG profile were all transient, with each returning to normoxic levels following systemic acclimation after 14 days of hypoxia.

A strength of our study is the use of a tightly-controlled rat model of hypoxic exposure, which shows physiological acclimation over a time course that has been defined previously [22]. Whilst the acute suppression of food intake upon hypoxic exposure can be a confounder in metabolic studies of this model [22], the inclusion of pair-fed normoxic animals in this study allowed us to separate the effects of a reduction in food intake from hypoxia per se. An additional strength of this study was the novel use of a CIII inhibitor with a well-understood toxicity profile [21, 23] in order to mechanistically investigate the role of the respiratory chain in the response to hypoxia.

We adopted multiple techniques to comprehensively assess the metabolic response to hypoxia, including targeted metabolomics and lipid profiling alongside detailed mitochondrial phenotyping. Our method for evaluating mitochondrial respiration utilises tissue homogenates, which can limit sensitivity in comparison with the use of isolated mitochondria. However, this approach allowed for swift preparation and minimised washout of the CIII inhibitor.

Within the 2 day period of hypoxic exposure, systemic adjustments to mitigate the fall in O_2_ delivery are yet to fully occur, and we saw no change in [Hb] within this timeframe. This period of acute hypoxic stress therefore represents a metabolic challenge for tissues such as liver. At this timepoint, enhanced OXPHOS capacity, potentially supported by an increase in mitochondrial supercomplex formation, occurs alongside downregulation of the oxygen-consuming process of DNL. This enhanced OXPHOS capacity appears to support metabolic homeostasis, with no change in expression of glycolytic enzymes or intermediates or of glycogen stores suggesting that at this timepoint there is no greater reliance on glycolytic flux for energetic homeostasis. Instead, suppression of the enhanced respiratory capacity via mild inhibition of CIII resulted in increased levels of glycolytic intermediates, underlining the importance of this enhanced capacity in maintaining oxidative metabolism. This therefore suggests enhanced respiratory capacity is necessary to prevent dependence upon glycolysis and maintain energetic homeostasis. This is in line with previous work in rodent heart and human skeletal muscle where metabolic adjustment to hypoxic exposure served to maintain tissue energetics [14, 45].

The increased OXPHOS capacity, and accompanying non-significant trend towards enhanced fatty acid oxidation capacity, does not fully compensate for the metabolic stress induced by acute hypoxia, with accumulation of hepatic long-chain acyl-carnitines indicating impaired fatty acid oxidation. Acyl-carnitines are derivatives of fatty acids required for transport across the inner mitochondrial membrane. Tissue acyl-carnitine levels are therefore indicative of flux through fatty acid oxidation and accumulation of long chain acyl-carnitines in hypoxia has previously been reported in the heart [46–48], plasma [46], and skeletal muscle [45]. Together with a decline in the expression of fatty acid metabolising enzymes [45, 49] and fatty acid oxidation capacity [14, 45, 50], this supports the notion of hypoxia-induced suppression of fat oxidation. Accumulation of acyl-carnitines may also result from decreased TAG synthesis, which has been associated with onset of lipotoxicity [51, 52].

The non-significant trend towards increased fatty acid oxidation capacity we observe after 2 day hypoxic exposure likely arose as a result of the acute suppression in food intake over this period. Indeed, there was a greater (and statistically-significant) increase in fatty acid oxidation capacity in rats pair-fed to match to the intake of hypoxic rats. This is in line with previous reports of elevated fatty acid oxidation in response to calorie restriction [53] and may result from activation of peroxisome-proliferator activated receptor α (PPARα) [54]. Calorie restriction has also been attributed to increasing ETS capacity upon the addition of FCCP [55, 56], an effect we too observed in the pair-fed animals and which was maintained after 2 days of hypoxia. In our study, hypoxia appears to partly suppress this acute increase in fatty acid oxidation that would otherwise be expected as a result of the fall in food intake, and this corresponds with the hypoxic suppression of PPARα transcriptional activity seen in other tissues [14, 22, 45]. Fatty acid oxidation is supported through electron transfer to complex I (via NADH) and the electron-transferring flavoprotein dehydrogenase (via free FADH_2_); however, complex II (succinate dehydrogenase) does also support fatty acid oxidation through its role in the TCA Cycle. The increase in S-Pathway flux that we observed following 2 days of hypoxia, which was not apparent in pair fed animals, could potentially impact on the capacity to oxidise fatty acids, alongside other oxidative substrates. However, the increase in S-pathway flux also appears to be insufficient to prevent long chain acyl-carnitine accumulation.

The findings of enhanced respiratory capacity, greater sensitivity to CIII inhibition, acyl-carnitine accumulation and other lipid profile changes occurring after 2 days of hypoxic exposure were transient, largely returning to normoxic levels following 14 days of hypoxic exposure. This suggests that acclimatisation alleviates the hypoxic stress through improvements in systemic O_2_ delivery. For instance, haemoglobin concentration was increased following 14 days of hypoxia, a response mediated by HIF-2α through induction of erythropoietin [57]. Whilst the present work does not delve further into the mechanisms of systemic acclimatisation that support hepatic metabolism with more sustained hypoxia, this would be an interesting avenue for future investigation.

Our examination of respiratory supercomplexes identified an increase in density of bands corresponding to supercomplexes I+III_2_ and III_2_+IV co-migrated with *V*_n_ following 2 days of hypoxic exposure, alongside factors critical for supercomplex assembly, cardiolipin and *Cox7a2l* expression. Each of these responses appeared to be unaffected by addition of the CIII inhibitor. This inhibitor has been shown to impair catalytic activity of CIII [21, 23], and our results suggest it does not impact supercomplex assembly. The aggregation of respiratory chain complexes into macromolecular assemblies are thought to increase efficiency of electron flow between complexes and promote complex stability [58]. Supercomplex assembly has been shown to dynamically adapt to changes in cellular metabolism [59]. This has been demonstrated in human skeletal muscle, with formation of complex I, III and IV containing supercomplexes decreasing in diabetic individuals [60], but increasing in response to exercise training [32]. In relation to hypoxia, sustained abundance of supercomplexes was found to be necessary to maintain robust growth of pancreatic ductal adenocarcinoma under conditions of extreme hypoxia (0.1% O_2_), a response dependent upon functional CIII and CIV [61]. Hypoxia is also known to affect the expression of *Higd1a*, a transcriptional target of HIF1α [62, 63], with increased levels being associated with cell survival in hypoxia [64]. In line with this, we found an increase in *Higd1a* expression following 2 days and 14 days of hypoxic exposure. However, given that the rise in supercomplex abundance was only observed after 2 day hypoxia, the sustained increase may link to other effects of *Higd1a* on respiratory chain performance. For instance, it has been shown to bind the heme α active center to ensure optimal activity of CIV in hypoxia [65] and to incorporate UQCRFS1 into CIII [66].

Our results demonstrate no change in supercomplex band density in response to reduced food intake over the 2 day period. The response to reduced calorie intake may be time-dependent, as the proportion of CIII assembled with CI has been shown fall in response to 18 h of starvation in mice [58].

Our examination of the hepatic lipid profile revealed a depletion of TAGs selectively enriched for palmitate, stearate, and oleate, which have been associated with DNL [44, 67]. This suggests suppression of DNL in response to the short-term hypoxic insult. DNL is the synthesis of fatty acid chains from acetyl CoA (AcCoA) derived from numerous metabolic reactions including glycolysis and amino acid deamination. These fatty acids subsequently undergo condensation with glycerol to form TAGs [68, 69]. Elongation of the substrate acyl chain ceases at the 16 or 18 carbon stage, with palmitic acid (C16) being the major product [70, 71]. Palmitic acid can be utilised to form a range of fatty acids, such as oleic acid through elongation to stearic acid followed by oxygen-dependent desaturation catalysed by stearoyl-CoA desaturase (SCD)1 [72, 73]. Examination of tumour-derived mammalian cell lines (MDA-MB-468, HeLa and A549) following 72 h in hypoxia (0.5–1% O_2_) revealed a decrease in SCD1 flux, a shift away from glucose-derived AcCoA towards glutamine-derived AcCoA, and an increase in fatty acid import [73]. Together, this implied DNL was bypassed in hypoxia [73].

An alternate hypothesis to explain the depletion of these specific hepatic TAG species at 2 days of hypoxia includes dietary changes. In humans, high protein and carbohydrate intake has been associated with inducing DNL [44, 74, 75], whilst calorie restriction has been reported to suppress hepatic DNL [75]. Here, we observed an increase in TAGs of chain lengths 39-53 in pair-fed rats, suggesting that the hypoxic driven changes in hepatic TAG composition are not due to decreased food intake. The profile of these TAG species may also be affected by alterations in adipose tissue turnover. Hypoxia has been associated with adipose tissue dysfunction, including impaired insulin-suppression of lipolysis [76], decreased expression of genes associated with DNL [77], and suppression of lipoprotein lipase activity alongside increased release of non-esterified fatty acids [78]. In rats, polyunsaturated fatty acids are the most abundant fatty acids and have the highest relative mobilisation rate from adipose tissue [79, 80], whereas monounsaturated and unsaturated fatty acids are only moderately mobilised [79, 80]. It is therefore unlikely that hypoxia induced changes in adipose tissue turnover affect incorporation of fatty acids 18:1, 18:0, 16:1, and 16:0 into TAGs as we observe.

An interesting avenue for future research would be the effects of high fat feeding on the hepatic hypoxic response. High fat feeding in rodents has been associated with the promotion of hypoxia signalling and mitochondrial dysfunction in liver [81], whilst a high-fat diet exacerbated energetic impairments in the hypoxic heart [14]. The combination of a high-fat, high-cholesterol diet and chronic intermittent hypoxia was associated with greater hepatic lipid peroxidation and inflammation [82]. The fatty acid composition of the diet used in any such manipulation would be of particular importance, for instance given that supplementation of oleic acid has been shown to protect against hepatic lipotoxicity induced by palmitic acid [83]. Dietary manipulation such as this may help to probe links between altered mitochondrial function and lipid metabolism at the hepatic and systemic level in hypoxia. This could be of particular importance when considering nutritional strategies for pathologies associated with systemic hypoxia, such as critical illness. Examination of skeletal muscle and plasma from critically ill patients has demonstrated evidence of lipid accumulation in the first 48 h following admission to an intensive care unit, alongside impairment of fatty acid oxidation, accumulation of muscle acyl-carnitines and diacylglycerol and plasma TAGs [84].

## Conclusion

In conclusion, mitochondria play a critical role in the hepatic response to shorter-term hypoxic stress, which is marked by a transient enhancement of respiratory capacity, associated with the formation of mitochondrial supercomplexes. This enhanced respiratory capacity is essential for certain aspects of metabolic homeostasis in the liver. Hepatic and systemic lipid metabolism is transiently disrupted by shorter-term hypoxia, with hypoxia-induced alterations to hepatic TAG profiles being sensitive to complex III inhibition. Thus, the liver responds to shorter-term hypoxia via changes in mitochondrial respiratory function and lipid metabolism.

## Methods

### Experimental model

Female Crl:CD(SD) rats (Charles River Laboratories) 220–300 g were randomly assigned one of 6 experimental groups (*n* = 8–10 per group). Female rats were chosen as the hepatotoxicity profile of GSK932121A is better understood in females than males [21]. The study design is presented in Fig. 1A. Rats were pair-housed in conventional cages in a temperature (23 °C) and humidity-controlled environment with a 12 h/12 h light/dark cycle. Rats were fed a standard diet (RM1(P), Special Diet Services, UK) and had access to food and water ad libitum, with the exception of the group pair-fed to match food intake of hypoxic animals as described below. Rats were randomly assigned either to remain under normoxic conditions (21% O_2_) or to be housed in hypoxia (10% O_2_) in a flexible-film chamber (PFI Systems Ltd., Milton Keynes, UK) for either 2 days or 14 days, with 20 air changes/h. Body mass, food and water intake were measured daily. At the end of the hypoxic exposure period, rats received an *i.p.* injection of either GSK932121A (a mitochondrial complex III inhibitor) or vehicle. The chemical structure of GSK932121A has been described previously [21]. The treatment comprised a nanomilled and spray-dried formulation of GSK932121A (49.5% w/w) containing mannitol (44.5% w/w), hydroxypropylmethylcellulose pharmacoat 603 (5% w/w), and sodium lauryl sulphate (1% w/w) suspended in sterile water. The vehicle was identical in formulation minus GSK932121A. A dosing concentration of 25 mg kg^−1^, expressed in terms of the parent compound, was administered.

Following administration, clinical signs were monitored every 15–30 min. The following were measured on a scale of 0–2, with 0 being not present, 1 obvious presentation, and 2 more severe presentation: degree of piloerection, hyperventilation, orbital tightening, and subdued behaviour [24]. The point of termination was determined by the severity of clinical signs and did not exceed 3 h post-injection. Rats were anaesthetised by *i.p.* injection of pentobarbital (Euthatal, Merial), at a dose of 500 mg kg^−1^ body mass. After cessation of peripheral sensitivity, the chest cavity was opened, and blood was collected from the left ventricle by cardiac puncture and transferred to an EDTA vacuette tube (K3 EDTA, Greiner Bio-one). A droplet was taken from this collection and used to measure blood glucose (Accu-Chek Compact Plus glucometer, Roche, Switzerland). Meanwhile, a droplet of blood taken from the tail vein was loaded into a microcuvette for quantification of haemoglobin concentration using a HemoCue Hb201 Analyzer (Ängelholm, Sweden).

The left lateral lobe of the liver was excised, and a portion placed into ice-cold biopsy preservation medium (BIOPS: 2.77 mM CaK_2_EGTA, 7.23 mM K_2_EGTA, 6.56 mM MgCl_2_.6H_2_O, 20 mM taurine, 15 mM phosphocreatine, 20 mM imidazole, 0.5 mM dithiothreitol, 50 mM MES, 5.77 mM Na_2_ATP, pH 7.1) for analysis by high-resolution respirometry. A further section was diced and placed into fixative for electron microscopy (EM). The remainder was snap-frozen in isopentane cooled on dry ice and stored at − 80 °C. All liver work described was performed on the left lateral lobe. To obtain plasma for metabolomics, whole blood collected was spun at 2000×*g* for 10 min at 4 °C and the plasma layer removed.

Animals pair-fed to match the food intake of hypoxic rats were also female Crl:CD(SD) rats (220–300 g) and were single-housed in normoxia (*n* = 6/group) in conventional cages in a temperature (23 °C) and humidity-controlled environment with a 12 h/12 h light/dark cycle, as described above. They were fed a standard diet (RM1(P), Special Diet Services, UK) and had access to water ad libitum. Rats were randomly assigned to a control group fed ad libitum or pair-fed to match the intake observed over 2 day hypoxic exposure: 10 g for the first 24 h, 14 g for the second 24 h. Termination and collection of the liver were performed as described above.

### Blood lactate

Within 30 min of blood collection, whole blood was added to tricarboxylic acid (0.6 N) at a ratio of 1:3 and vortexed thoroughly. Blood lactate was assessed as described previously [21].

### Ultraperformance liquid chromatography (UPLC) for GSK932121A detection

Snap-frozen liver (100–200 mg) was homogenised using Precellys Tissue Homogeniser (Bertin Instruments) in water to give a 1:3 (w/v) sample using 3 × 30 s cycles at 6500 rpm. For extraction, 10 μl of sample was added to internal standard working solution (ISWS, 10 ng/mL [^13^C_6_]-GSK932121A in Acetonitrile), vortexed and centrifuged for 15 min at 3000*g*. Snap frozen plasma (50 μl) was extracted using 25 ng/mL ISWS. UPLC analysis was performed using AQUK35 (Waters Acquity) UPLC system (Agilent, United States) coupled with UK22-AB/Sciex API4000 (Sciex, USA) using Positive-ion TurboIonSpray® (Sciex). The assay was run over 1.5 min using a 50 × 2.1 mm BEH C18 1.7 μm column (Waters^TM^ Acquity) conditioned at 50 °C with a flow rate of 0.8 mL/min and 0.5 μl of sample. The mobile phase consisted of (A) HPLC grade water, 0.1% formic acid, and (B) Acetonitrile. GSK932121A analyte precursor and product ions were detected at 426.1 m/z and 231.1 m/z respectively at 1 min retention time. Analysis was performed using Analyst 1.4.1 (Sciex).

### Liver glycogen

Snap-frozen liver was prepared and analysed using a glycogen assay kit (Abcam, ab65620) following manufacturer’s instructions. Values were corrected to protein concentration obtained from a bicinchoninic acid (BCA) assay (BCA1-1KT, Sigma).

### Liver high-resolution mitochondrial respirometry

The BIOPS-preserved liver sample was prepared for respirometry as described previously [85]. Respiration rates were analysed using a substrate-uncoupler inhibitor titration in duplicate on each biological replicate. *J*_O2_ was measured following the addition of octanoyl-carnitine (0.2 mM) and malate (1 mM) initially in the LEAK state, i.e., without ADP (OctM_*L*_), and then in the OXPHOS state following the addition of ADP (10 mM) (OctM_*P*_). The N-pathway via complex I was then stimulated with the addition of glutamate (10 mM) (GM_*P*_), followed by cytochrome *c* (10 μM) to assess outer mitochondrial membrane integrity. Maximal OXPHOS was then stimulated with addition of succinate (10 mM) (GMS_*P*_). Stimulation of maximal electron transfer system (ETS) capacity was achieved through the addition of carbonyl cyanide-p-trifluoromethoxyphenylhydrazone (FCCP) (titrated in 0.5 μM additions until maximal capacity was reached) (GMS_*E*_). Finally, complex I was inhibited through the addition of rotenone (0.5 μM), restricting electron flux to the S-pathway via complex II (S_*E*_). Respiration rates were normalised to wet mass of liver tissue.

### Citrate synthase activity

Snap-frozen liver was prepared and analysed for citrate synthase activity as described previously [86].

### Metabolomics/lipidomics

A chloroform/methanol extraction was performed on snap-frozen liver (~ 30 mg) and plasma (20 μl) as described previously [45] followed by ultra-high performance liquid chromatography mass spectrometry [87, 88].

The aqueous phase underwent normal and reverse phase analysis. The aqueous and organic fractions were combined for carnitine analysis. The protein pellet was re-suspended in RIPA buffer (ThermoScientific) containing protease inhibitor (Roche) and the protein concentration determined using a BCA assay (BCA1-1KT, Sigma). Data were processed using the vendor’s software and normalised to protein concentration and to the intensity of internal standards.

### Aqueous metabolite analysis

Reverse phase analysis was performed as described previously [88]. Before the analysis, samples were reconstituted in 0.1 mL of a 10 mM ammonium acetate water solution containing a mixture of 8 internal standards at the concentration of 10 μM (D3 - proline, D8-valine, D10-leucine, U-^13^C lysine, ^13^C-glutamic acid, D5-phenylalanine and, D3-succinic acid and D4- serotonin). Normal phase analysis was performed using a Thermo Scientific Vanquish^TM^ UHPLC^+^ series coupled with a TSQ Quantiva mass spectrometer (Thermo Fisher Scientific, Waltham, MA, USA) and was used with an electron spray ionisation (ESI) source, operated in positive and negative ion mode with polarity switching. The electrospray voltage was set to 3500 V for the positive ionisation and to 2500 V for the negative ionisation. N_2_ at 48 mTorr and 420 °C was used as a drying gas for solvent evaporation. The aqueous phase was analysed with a BEHAmide (150 × 2.1 mm 1.7 μm) column. The column was conditioned at 30 °C. The mobile phase consisted of: (A) a 0.1 M of aqueous solution of ammonium carbonate and (B) acetonitrile. The mobile phase was pumped at a flow rate of 600 μL/min programmed as follows: initially at 20% of A for 1.5 min, then subjected to a linear increase from 20% to 60% of A over 2.5 min and kept at this percentage for 1 min before being brought back to initial conditions after 0.1 min, followed by 3 min of equilibration. Xcalibur software (Thermo Fisher Scientific version 4.1, Waltham, MA, USA) was used for data acquisition. Putative recognition of all detected metabolites was performed using a targeted MS/MS analysis. Before the analysis, samples were reconstituted in 0.1 mL of acetonitrile: 1 M aqueous ammonium carbonate solution (7:3 v/v) containing a mixture of 3 internal standards at the concentration of 10 μM (^13^C-glutamic acid, D3-succinic acid and AMP).

### Carnitine analysis

Samples were prepared as described previously [45, 85]. A Thermo scientific UHPLC^+^ series coupled with a TSQ Quantiva mass spectrometer (Thermos fisher scientific, Waltham, MA, USA) and was used with an ESI source, operated in positive and negative ion mode at the same time. The electrospray voltage was set to 3500 V for the positive ionisation and to 2500 V for the negative ionisation. N_2_ at 48 mTorr and 420 °C was used as a drying gas for solvent evaporation. The combined aqueous and organic phases were analysed with an ACE Excel 2 C18 PFP (100A. 150 × 2.1 mm 5 μm) column. The column was conditioned at 30 °C. The mobile phase consisted of (A) 0.1% of formic acid water solution and (B) methanol solution. The mobile phase was pumped at a flow rate of 0.450 μL/min programmed as follows: initially stayed at 0.5% of B for 1 min, then subjected to a linear increase from to 100% of A over 9 min and kept at this percentage for 2 min before being brought back to initial conditions after 0.1 min. Xcalibur software was used for data acquisition. Putative recognition of all detected metabolites was performed using a targeted MS/MS analysis. Before the analysis, the combined carnitine fraction was reconstituted in 0.1 mL of a methanol: water solution (4:1 v/v) containing an internal standard mix of eight deuterated carnitines at a concentration of 2 μM (free carnitine, C2, C3, C4, C5, C8, C14, C16).

### Lipidomics

A chloroform/methanol extraction was performed, as stated above for liver, as well as on 20 μl snap frozen plasma. For both liver and plasma, the dried organic fraction was reconstituted in 50 μL of methanol/chloroform (1:1) and vortexed thoroughly. Ten microliters of the sample was then diluted into 190 μL of isopropyl alcohol/acetonitrile/water (2:1:1) and briefly vortexed.

An LTQ Orbitrap Elite Mass Spectrometer (Thermo Fisher Scientific) was used in positive and negative modes. Metabolites were ionised by heated electrospray before entering the spectrometer. The source temperature was set to 420 °C, and the capillary temperature to 380 °C. In positive mode, the spray voltage was set to 3.5 kV, whilst in negative it was 2.5 kV. Data was collected using the Fourier transform mass spectrometer (FTMS) analyser. The resolution was set to 60,000 and the data was obtained in profile mode. The full scan was performed across an m/z range of 110–2000. For both modes, 5 μL of sample was injected onto a C18 CSH column, 2.1 × 50 mm (1.7 μM pore size) (Waters), which was held at 55 °C using an Ultimate 3000 UHPLC system (Thermo Fisher Scientific). The mobile phase comprise solvents A (acetonitrile/water 60:40) and B (acetonitrile/isopropanol 10:90), run through the column in a gradient (40% B, increased to 43% B after 0.8 min, 50% B at 0.9 min, 54% B at 4.8 min, 70% B at 4.9 min, 81% B at 5.8 min, raised to 99% B at 8 min for 0.5 min before returning to 40% for 1.5 min). Total run time was 10 min, with a flow rate of 0.500 μL/min. In positive mode, 10 mM ammonium formate was added to solvents A and B. In negative mode, 10 mM ammonium acetate was the solvent additive. Solvent additives were chosen based on previous work [89]. Before analysis, 250 μl internal standard (IS) mix was added to each sample. This was composed of deuterated standards sourced from Avanti Polar Lipids (C16-d31 Ceramide, 16:0-d31-18:1 PA,16:0-d31-18:1 PC, 16:0-d31-18:1 PE, 16:0-d31-18:1 PG, 16:0-d31-18:1 PI,14:0 PS-d54, and 16:0-d31 SM) and CDN Isotopes/QMX Laboratories (18:0-d6 CE, 15:0-d29 FA, 17:0-d33 FA, 20:0-d39 FA, 14:0-d29 LPC-d13, 45:0-d87 TG, 48:0-d83 TG, and 54:0-d105 TG). IS mix was made in 1:1 methanol chloroform, and each standard was at 2.5 μg/mL.

For processing, spectra were converted to .mzML files using MSConvert (Proteowizard) for subsequent analysis. XCMS software within R was used to process data and identify peaks. Peaks were identified based on an approximate FWHM of 5 sec and a signal-to-noise threshold of 5. To improve peak identification, peaks had to be present in a minimum of 25% of the samples. Peaks were annotated by accurate mass and retention time using an in-house R script and comparison to the LipidMaps database [90]. Peak intensity was normalised to internal standards and, in the instance of liver, to protein concentration.

### Fragmentation analysis

Triacylglycerol chain composition was analysed using a data-dependent acquisition (DDA)-based fragmentation step. Ions were fragmented using collision induced dissociation (CID) at a normalised collision energy of 35. Precursor ions were selected from a mass list, with the most intense ion on the list fragmented in each scan. The minimum signal required was 5000 counts and the isolation width set to 1. The activation time was 10 ms and the activation Q set to 0.25. Fragmentation spectra were acquired in centroid mode at a resolution of 15,000 using the FTMS analyser. Lipid identity was determined through manual identification of fragmentation patterns according to published methods [91], a process aided by use of an online resources http://www.byrdwell.com/Triacylglycerols/.

For both positive and negative ionisation modes, 5 μL of sample was injected onto a C18 CSH column, 75 μM × 100 mm (Waters, 186005297), which was held at 55^o^C using an Ultimate 3000 UHPLC system (Thermo Fisher Scientific). The mobile phase comprise solvents A (acetonitrile/water 60:40) and B (acetonitrile/isopropanol 10:90), run through the column in a gradient (40% B, increased to 43% B after 2 min, 50% B at 2.1 min, 54% B at 12 min, 70% B at 12.1 min, raised to 99% B at 18 min before returning to 40% for 2 min). Total run time was 20 min, with a flow rate of 0.400 μL/min. In positive mode, 10 mM ammonium formate (Fisher Scientific, A/3440/53) was added to solvents A and B. In negative mode, 10 mM ammonium acetate (Sigma Aldrich, 516961) was the solvent additive.

### Transmission electron microscopy

Samples of liver were fixed in 4% Formaldehyde/1% Glutaraldehyde, washed in Millonig’s Phosphate buffer and transferred to a Leica EMTP automatic tissue processor (Leica Microsystems). Tissues were post-fixed in 2% Osmium Tetroxide and processed into Agar 100 Epoxy resin (Agar Scientific). Samples were embedded and allowed to polymerise overnight at 60 °C. From the resulting blocks semi-thin (1 μM thick) sections were cut on a Leica UC6 Ultra-microtome (Leica Microsystems) and stained with Toluidine Blue to locate the correct region using light microscopy. Subsequently Ultra-thin (80-90 nm thick) sections were cut and contrasted using 2% Lead Citrate and Uranyless stain (TABB). Grids were examined using a Hitachi H7500 Transmission Electron Microscope (Hitachi High-tech) operating at 80 kV and images taken on a Gatan OneView Digital Camera (Gatan, Inc.). Mitochondrial number was quantified in images taken at × 7000 magnification through a manual count, corrected to image area.

### Blue native polyacrylamide gel electrophoresis (BN-PAGE)

Snap-frozen liver samples (~ 20 mg) were prepared and BN-PAGE performed as previously described [31] with the addition of NativeMark^TM^ unstained protein standard (Invitrogen, LC0725). Following BN-PAGE, the gel was stained using a Colloidal Blue Staining kit (Invitrogen, LC6025) as per manufacturer’s instructions. The gel was then washed overnight in ddH_2_O prior to imaging using HP Scanjet G4050 and analysis using ImageJ software [92]. Band density was corrected to CII, as described previously [60]. Band identification was based on immunoblotting and comparison to prior work utilising this approach in rodent liver [31]. Immunoblotting was performed after transfer of unstained gels, as previously described [31]. After 50 minutes incubation in blocking buffer (5% Bovine Serum Albumin (Sigma, A6003) TBS-T), staining of the individual complexes was achieved through overnight incubation at 4 °C with one of the following primary antibodies diluted in blocking buffer: NDUFA9 (Invitrogen, Cat#459100, RRID:AB_2532223, 2:1000), SDHA (Invitrogen, Cat#459200, RRID:AB_2532231, 1:10,000), UQCRC2 (Abcam, ab14745, RRID: AB- 2213640 1:1000) MTCO1 (Abcam, ab14705, RRID:AB_2084810, 1:1000), and ATP5A (Abcam, ab14748 RRID:AB_301447, 1:1000). The membrane was incubated with secondary antibody (Rabbit anti-Mouse IgG HRP, #61-6520, Invitrogen; RRID:AB_2533933 1:10,000 in TBS-T)) for 1 h at room temperature, before ECL detection (Milipore) and imaging using iBright 1500 (ThermoFisher Scientific).

Anti-OXPHOS antibody cocktail (Thermo Fisher Scientific, Cat#45-8099, RRID:AB_2533835) was also used, followed by secondary antibody as above and staining using Immobilon^TM^ Western Chemiluminescent HRP Substrate (Millipore). To gain clearer resolution of the CII band, the membrane was washed overnight using Tris-buffered saline (TBS) –Tween and incubated with SDHA monoclonal antibody (Invitrogen, Cat#459200, RRID:AB_2532231, 1:10,000).

Original immunoblot images for BN-PAGE gels are presented in Additional File 1: Figure S8. Original colloidal blue staining images are presented in Additional File 1: Figure S9.

### Immunoblotting for OXPHOS complexes

Preparation of tissue lysate was carried out as described previously [93]. From this, 10 μg protein was loaded into 4–20% gradient gels (Mini-PROTEAN® TGX™ Precast Protein Gels, 15-well, 15 μl #4561096, BioRAD) alongside a protein ladder (Precision plus protein dual color standards #1610374, BioRAD). The gel was transferred to nitrocellulose membrane, and this was stained in Ponceau S. The membrane was blocked in 5% Skimmed milk-TBS-T for 1 h prior to primary antibody incubation (Total OXPHOS antibody cocktail, ab110412, Abcam, RRID:AB_2847807; 1:500 in 1% Milk TBS-T) overnight at 4 °C. The membrane was incubated with secondary antibody (Rabbit anti-Mouse IgG HRP, #61-6520, Invitrogen; RRID:AB_2533933 1:5000 in TBS-T)) for 1 h at room temperature, before ECL detection (Milipore) and imaging using iBright 1500 (ThermoFisher Scientific). Band density was quantified using Image J software [92]. The original immunoblot image is presented in Additional File 1: Figure S8H.

### Gene expression analysis

Total RNA was extracted from frozen liver (~ 20 mg) using a RNeasy Plus Universal Mini Kit (QIAGEN), as per manufacturer’s instructions. The concentration of eluted RNA was measured using a Nanodrop DN-1000 spectrophotometer. cDNA synthesis was carried out using the qScript synthesis kit, following the manufacturer’s protocol (Quantabio). mRNA expression was measured by quantitative (Q)-PCR using SYBR Green Mastermix (Eurogentec Ltd.) and the DNA Engine Opticon 2 system (BioRad). Primers were obtained from QuantiTech Primer Assay (QIAGEN) and product details are as follows: Rn_Hk2_1 QT00190764, Rn_Pfkl_1 QT00175651, Rn_Ldha_2 QT02336243, Rn_Higd1a_1 QT00372428, Rn_Stoml2_1 QT01571724 , Rn_Slc25a11_1 QT01082914 , Rn_Actb_1 QT00193473. In the instance of Cox7a2l, QuantiFast SYBR Green PCR kit (QIAGEN) and QuantStudio 1 Real-Time PCR System (Applied Biosystems, Thermo Fisher) were used. The primer was obtained from QuantiTect Primer Assays (QIAGEN) with the following product details: Rn_Cox7a2l_1_SG. In all cases, transcript levels were normalised to levels of *Actb* and fold change determined using the 2^−ΔΔCT^ method, with expression in vehicle/normoxic animals normalised to 1.

### Quantification and statistical analysis

For comparisons between the effects of hypoxia and GSK93121A treatment, a two-way ANOVA was employed. In the instance of comparison between hypoxic treatment alone, a one-way ANOVA was employed. Where significant differences were found, post-hoc pairwise comparisons were carried out with a Tukey’s correction.  Regression analysis was performed using simple linear regression. Analysis was carried out using GraphPad Prism 8 software (GraphPad Software Inc.), and differences were considered significant when *p* ≤ 0.05. Where bar charts are used, data are presented as ± SEM.

In the instance of targeted metabolomics (aqueous fraction and carnitines), values were corrected to internal standard and tissue protein concentration prior to ANOVA testing. A false discovery rate correction was also employed (two-stage linear step-up procedure of Benjamini, Krieger, and Yekutieli, *Q* = 5%, threshold *p* value ≤ 0.032) to account for application of multiple ANOVAs. A post-hoc Tukey’s test was performed following this to define significant interactions. For presentation in heatmaps, data was normalised using autoscaling and generalised logarithm transformation using Metaboanalyst [94].

For open-profile lipidomic analysis, multivariate analysis was adopted. Data was first normalised by Pareto scaling and generalised logarithm transformation using Metaboanalyst. The strategy for this analysis was informed by the metabolomics and respirometry, which indicated a marked metabolic effect occurring following 2 days of hypoxic exposure. Lipid profiles were compared using orthogonal partial least squares—discriminant analysis (OPLS-DA), carried out using SIMCA (version 15, Umetrics, Umea, Sweden). The discriminants driving the separation in profiles were defined as those lying 2 SD away from the mean on an S plot. For presentation in heatmaps, normalised data was used. Percentage changes and related statistical testing were calculated from original peak intensity data using either one-way ANOVA when comparing hypoxic effect only, or two-way ANOVA when comparing hypoxic and GSK932121A-treatment effects. GraphPad Prism was used for this analysis and data presentation.

For analysis of the effects of pair-feeding to match that consumed by the hypoxic animals, an unpaired Student *t* test was employed to compare the control to the pair-fed.

## Supplementary Information

**Additional file 1: Figure S1.** Systemic and hepatic effects of GSK932121A administration. **Figure S2.** Food intake and body weights for Veh and GSK932121A treated animals, alongside hepatic mitochondrial respiration rates from control and hypoxic pair-fed animals. **Figure S3.** BN-PAGE band identification and band intensity quantification for control and hypoxic pair-fed animals. **Figure S4.** Protein expression of mitochondrial respiratory chain complexes and gene expression of factors associated with mitochondrial supercomplex formation. **Figure S5.** Glycolytic gene expression. **Figure S6.** Effect of hypoxia pair-feeding on hepatic TAGs. **Figure S7.** Lipidomic profile of 2 d hypoxic Veh and GSK932121A treated animals, TAG levels with GSK932121A treatment and total lipid intensity. **Figure S8.** Original immunoblot images. **Figure S9.** Original colloidal blue stained images obtained using BN-PAGE.

## Data Availability

The datasets supporting the results presented in this article are freely available via the Cambridge University Repository: 10.17863/CAM.75680 [95]. Raw metabolomics data have been deposited to the EMBL-EBI MetaboLights database [96] with the identifier MTBLS3713. The complete dataset can be accessed here: https://www.ebi.ac.uk/metabolights/MTBLS3713 [97].

## Abbreviations

BN-PAGE: Blue native polyacrylamide gel electrophoresis
CPT1: Carnitine palmitoyl transferase 1
CI - V: Respiratory chain complexes I-V
DNL: De novo lipogenesis
ETS: Electron transfer system
Hb: Haemoglobin
HIF: Hypoxia inducible factor
LC-MS: Liquid chromatography –mass spectrometry
OPLS-DA: Orthogonal partial least squares – discriminant analysis
OXPHOS: Oxidative phosphorylation
PPARα: Peroxisome proliferator-activated receptor α
TAG: Triacylglyerol
TCA: Tricarboxylic acid
UPLC: Ultra performance liquid chromatography
Veh: Vehicle treatment
