# Supplementary material for: Enhanced hepatic respiratory capacity and altered lipid metabolism support metabolic homeostasis during short-term hypoxic stress
Source: BMC Biol. 2021 Dec 15;19:265. doi: 10.1186/s12915-021-01192-0 (PMC8675474; doi:10.1186/s12915-021-01192-0)
Supplement: Supplementary file 1 — Additional file 1: Figure S1. Systemic and hepatic effects of GSK932121A administration. Figure S2. Food intake and body weights for Veh and GSK932121A treated animals, alongside hepatic mitochondrial respiration rates from control and hypoxic pair-fed animals. Figure S3. BN-PAGE band identification and band intensity quantification for control and hypoxic pair-fed animals. Figure S4. Protein expression of mitochondrial respiratory chain complexes and gene expression of factors associated with mitochondrial supercomplex formation. Figure S5. Glycolytic gene expression. Figure S6. Effect of hypoxia pair-feeding on hepatic TAGs. Figure S7. Lipidomic profile of 2 d hypoxic Veh and GSK932121A treated animals, TAG levels with GSK932121A treatment and total lipid intensity. Figure S8. Original immunoblot images. Figure S9. Original colloidal blue stained images obtained using BN-PAGE. [file 12915_2021_1192_MOESM1_ESM.pdf]

Additional File 1: Fig S1

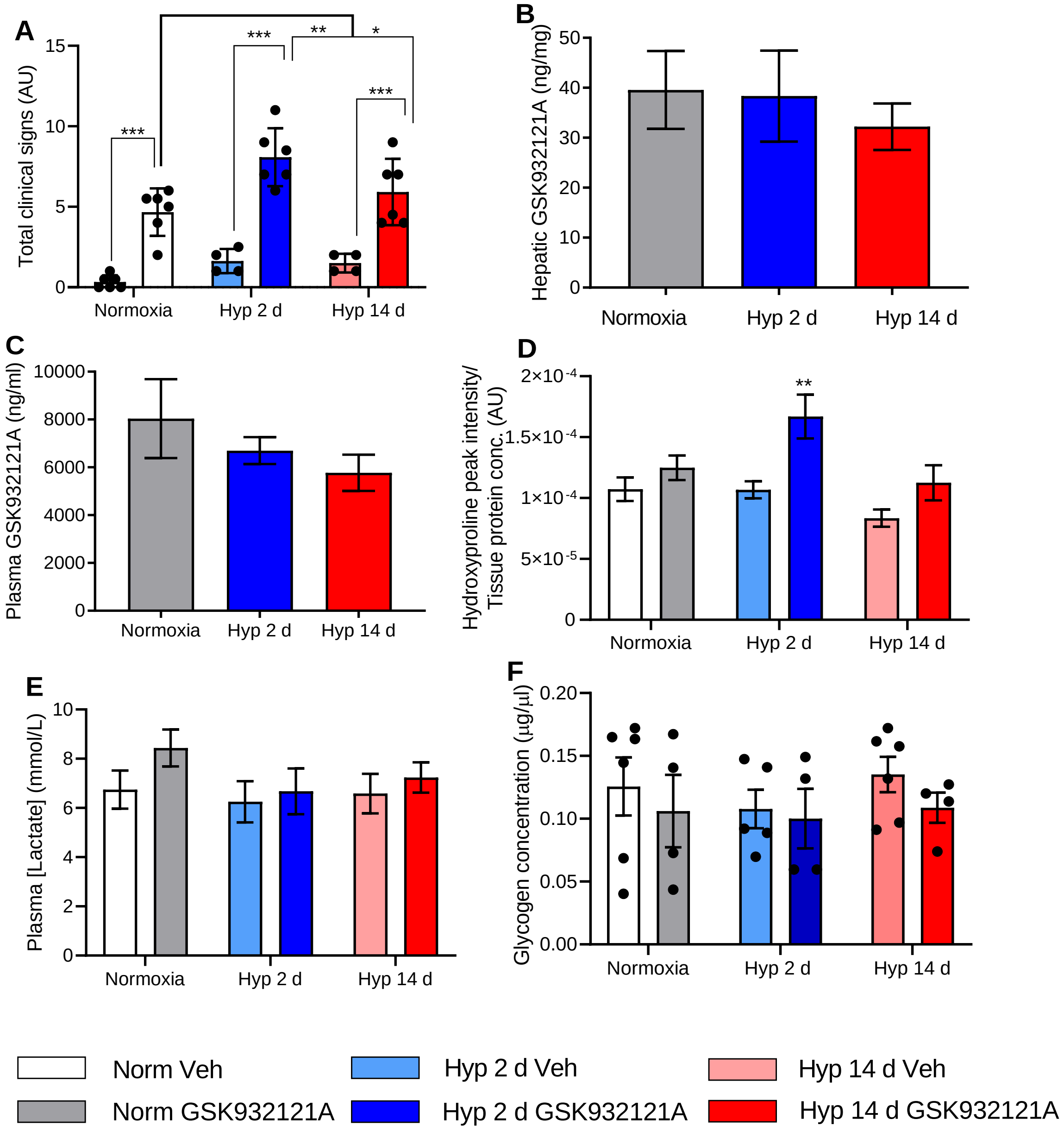

Additional File 1: Fig S1. Systemic and hepatic effects of GSK932121A administration.

A. Total clinical signs including degree of piloerection, hyperventilation, orbital tightening and subdued behaviour, each measured on a scale of 0-2. n = 6 Veh normoxic, n = 4 for Veh 2 d and 14 d Hyp, n = 6 for all GSK932121A treated groups.

B. Hepatic CIII inhibitor concentration, measured by ultra-performance liquid chromatography-mass spectrometry (UPLC) and expressed as ng/mg, n = 8 per group.

C. Plasma CIII inhibitor concentration, measured by UPLC and presented as ng/mL, n = 8 per group.

D. Hepatic hydroxyproline content, measured by LC-MS presented as peak intensity corrected to an appropriate internal standard and sample liver tissue protein concentration. n = 9 Veh normoxic, n = 8 for all remaining groups.

E. Plasma lactate concentration expressed as mmol/L. n = 10 Veh normoxic, n = 8 for all remaining groups.

F. Hepatic glycogen concentration expressed as µg/µl. n = 6 Veh normoxic and 14 d hypoxic, n = 5 Veh 2 d hypoxic, n = 4 for all GSK932121A treated groups.

All data presented as mean ± SEM, * $p < 0.05$, ** $p \leq 0.01$, *** $p \leq 0.001$, **** $p \leq 0.0001$.

Additional File 1: Fig S2

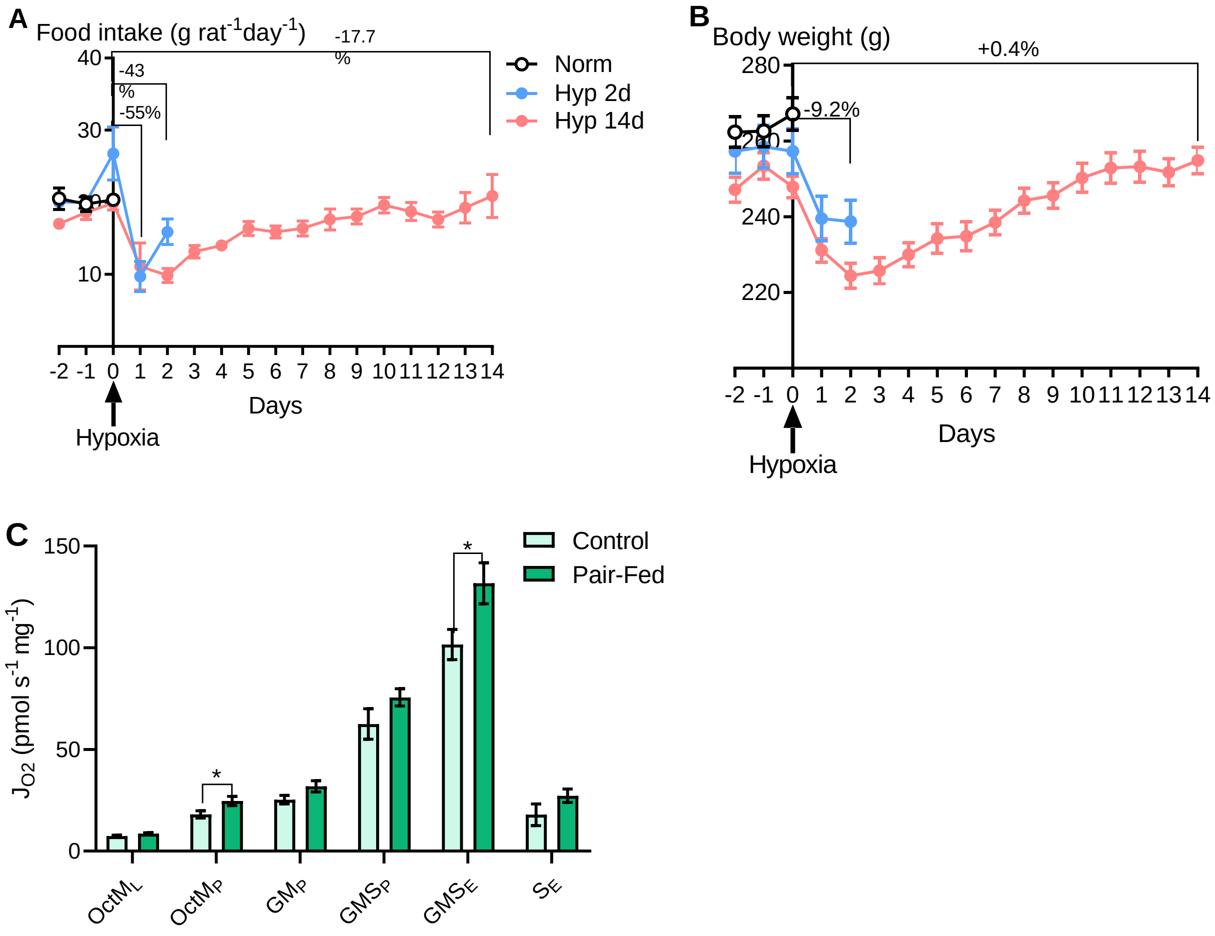

<u>Additional File 1: Fig S2.</u> **Food intake and body weights for Veh and GSK932121A treated animals, alongside hepatic mitochondrial respiration rates from control and hypoxic pair-fed animals.**

A. Food intake (g) per rat per day, vehicle and GSK932121A-treated groups combined, n = 9 normoxic, n = 8 for all remaining groups.

B. Body weight measured daily. Vehicle and GSK932121A-treated groups combined, n = 18 normoxic, n = 18 for all remaining groups, is presented as mean ± SEM.

C. Respiration rates of liver homogenates from control (light green) and hypoxia pair-fed (dark green) rats across the following states: Octanoyl carnitine and malate-supported (β-oxidation) respiration without ADP ($OctM_L$) and with ADP ($OctM_P$). Stimulation of complex I-supported respiration with the addition of glutamate (GM_P) and stimulation of maximal OXPHOS following the addition of succinate (GMS_P). Stimulation of maximal electron transfer system (ETS) capacity through titration of FCCP (GMS_E), and finally inhibition of complex I through the addition of rotenone, restricting electron flux through complex II (S_E). Respiration rates are corrected to liver tissue mass added to the chamber and are presented as ± SEM, n = 6 per group, * $p < 0.05$.

Additional File 1: Fig S3

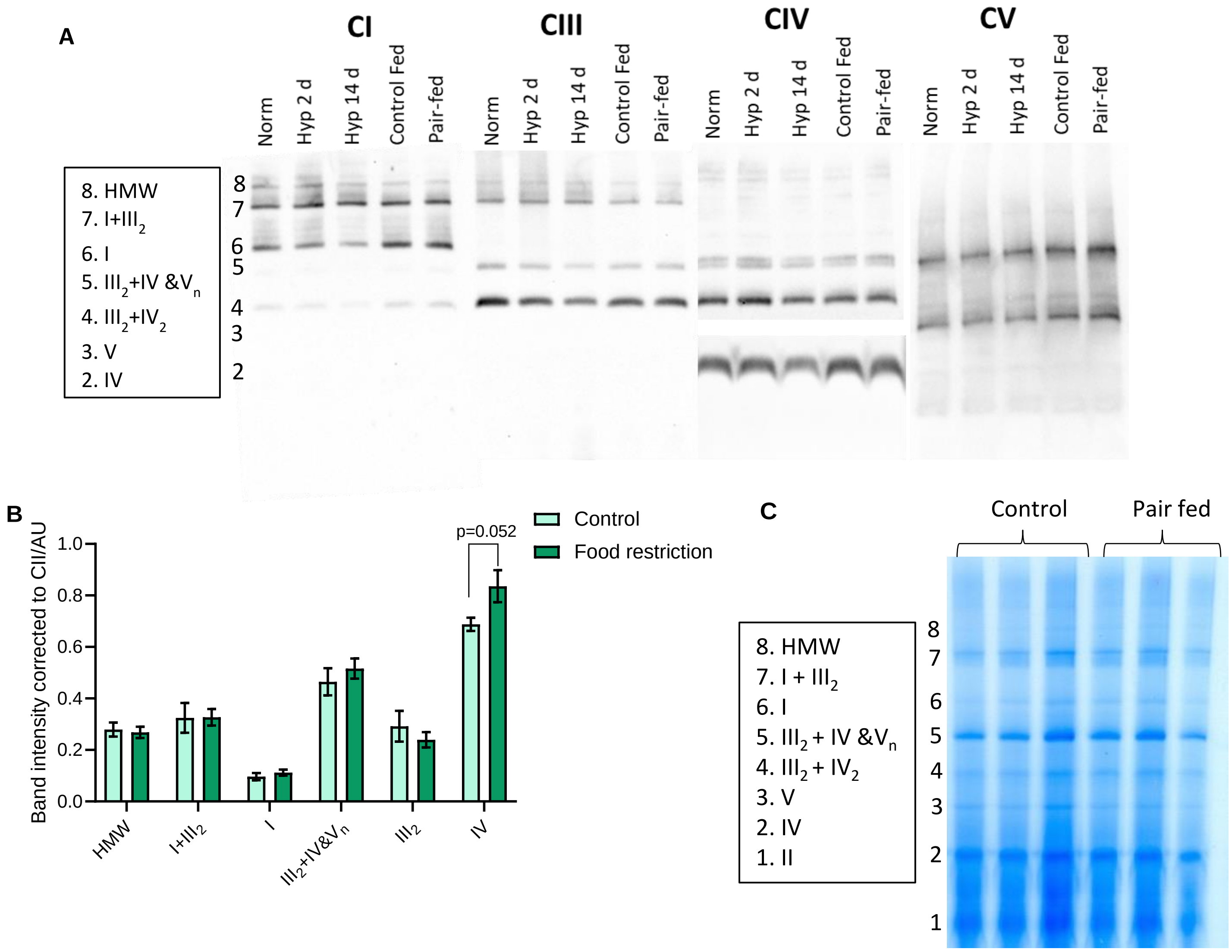

<u>Additional File 1: Fig S3.</u> BN-PAGE band identification and band intensity quantification for control and hypoxic pair-fed animals.

A. Example immunoblots across experimental groups from BN-PAGE gels for respiratory complexes I,III, IV, V to confirm band identity. To avoid over-saturation of the bottom band, the Complex IV immunoblot is presented as two separate exposures.

B. Band intensity for the following mitochondrial complex and supercomplex stoichiometric combinations in control and pair-fed animals: higher molecular weights (HMWs), I+III_2, III_2+IV and V_n; alongside mitochondrial complexes IV and I. All are corrected to complex II levels. Presented as mean ± SEM, n = 6 per group.

C. Colloidal blue staining of a representative gel from BN-PAGE analysis of control and pair-fed animals, from which band density was quantified.

<u>**Additional File 1: Fig S4.**</u>

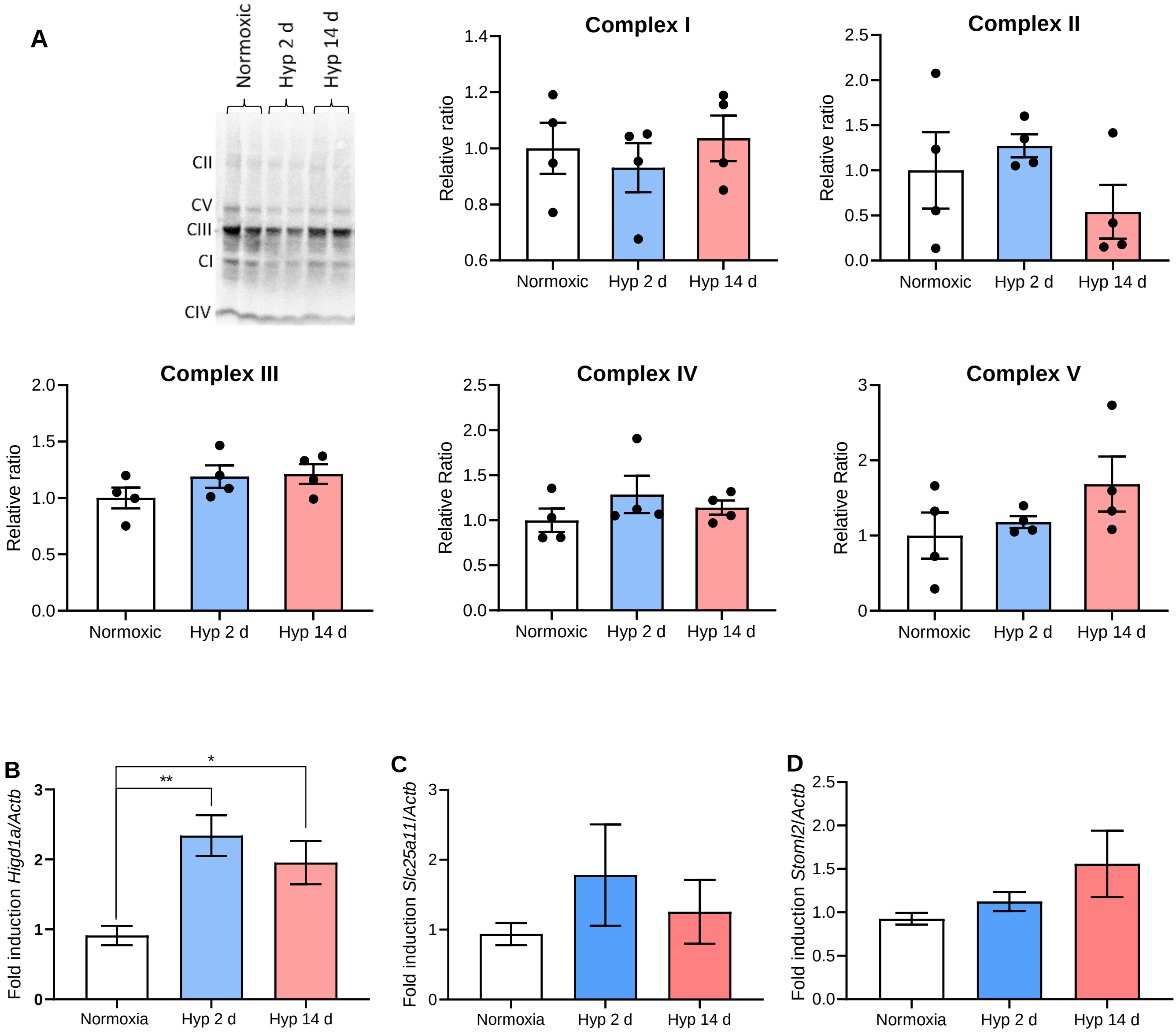

<u>Additional File 1: Fig S4.</u> Protein expression of mitochondrial respiratory chain complexes and gene expression of factors associated with mitochondrial supercomplex formation.

A. Western blot image and analysis for expression of mitochondrial respiratory complexes I-V. Data is corrected to ponceau and loading control and is expressed as relative ratio to normoxic control. Veh-treated and GSK932121A dosed animals combined, n = 4 per group.

B-D. Expression of the genes encoding HIGD1A (B), SCL25A11 (C) and STOML2 (D) proteins measured using qPCR and presented as fold induction corrected to *Actb* expression. Data for (A) includes Veh and GSK932121A dosed animals combined, n= 15 normoxic and hypoxic 14 d, n = 17 for 2 d hypoxic. Data for (C) and (D) includes Veh-treated animals , n = 7 normoxic, n = 8 hypoxic. Data is presented as mean ± SEM, * $p < 0.05$, ** $p \leq 0.01$.

Additional File 1: Fig S5.

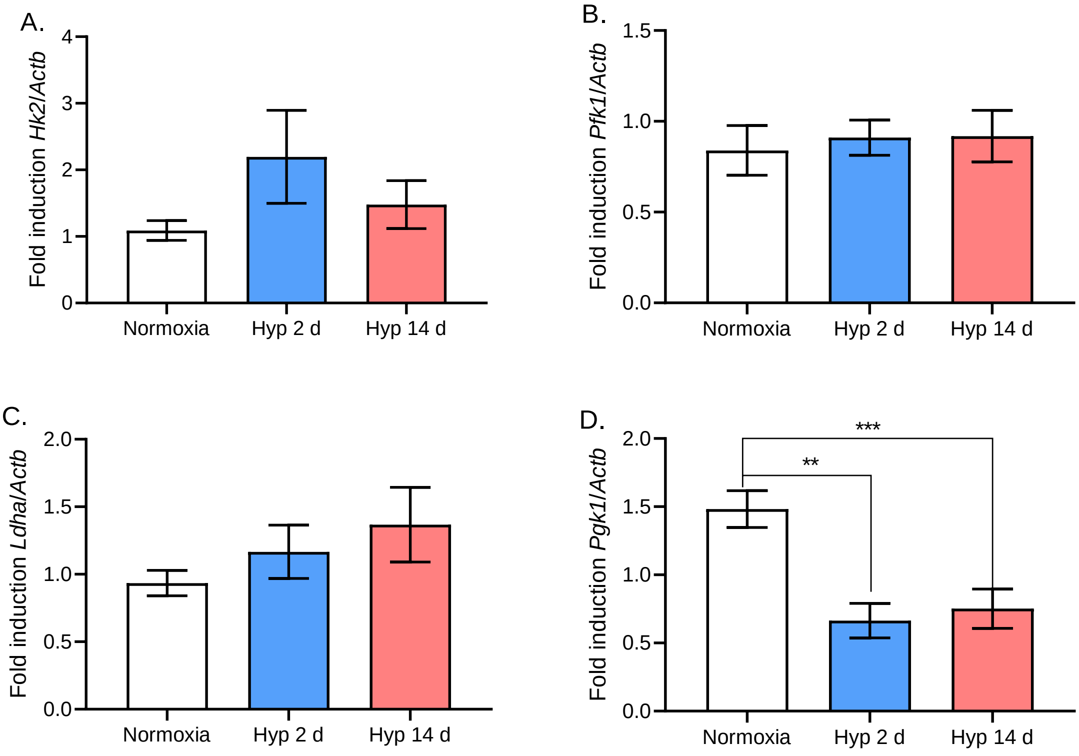

<u>Additional File 1: Fig S5.</u> Glycolytic gene expression.

A-D. Fold induction of glycolytic genes in Veh-treated animals: *Hk2, Pfk1, Ldha, Pgk1.* Assessed using qPCR, corrected to *Actb* expression and levels in Veh-treated normoxic rats. All data presented as mean ± SEM, ** $p \leq 0.01$, *** $p \leq 0.001$. n = 8 for normoxic and 2 d hypoxic, n = 6 for 14 d hypoxic (A, D), n = 7 for 14 d hypoxic (B,C).

Additional File 1: Fig S6.

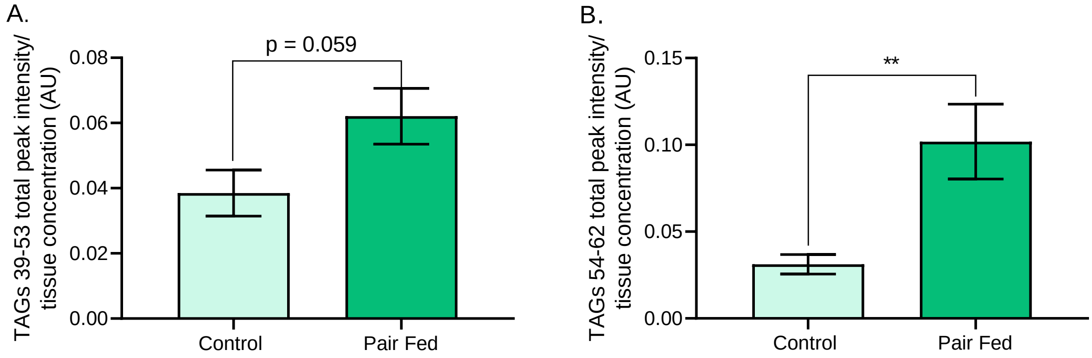

<u>Additional File 1: Fig S6.</u> Effect of hypoxia pair-feeding on hepatic TAGs.

A. Total peak intensities of triacyglycerols (TAGs) with carbon chain lengths 39-53 in control and pair fed animals
B. Total peak intensities of TAGs with chain lengths 54-62 in control and pair fed animals.

Data presented as mean ± SEM, ** $p < 0.01$, n=6 per group

Additional File 1: Fig S7.

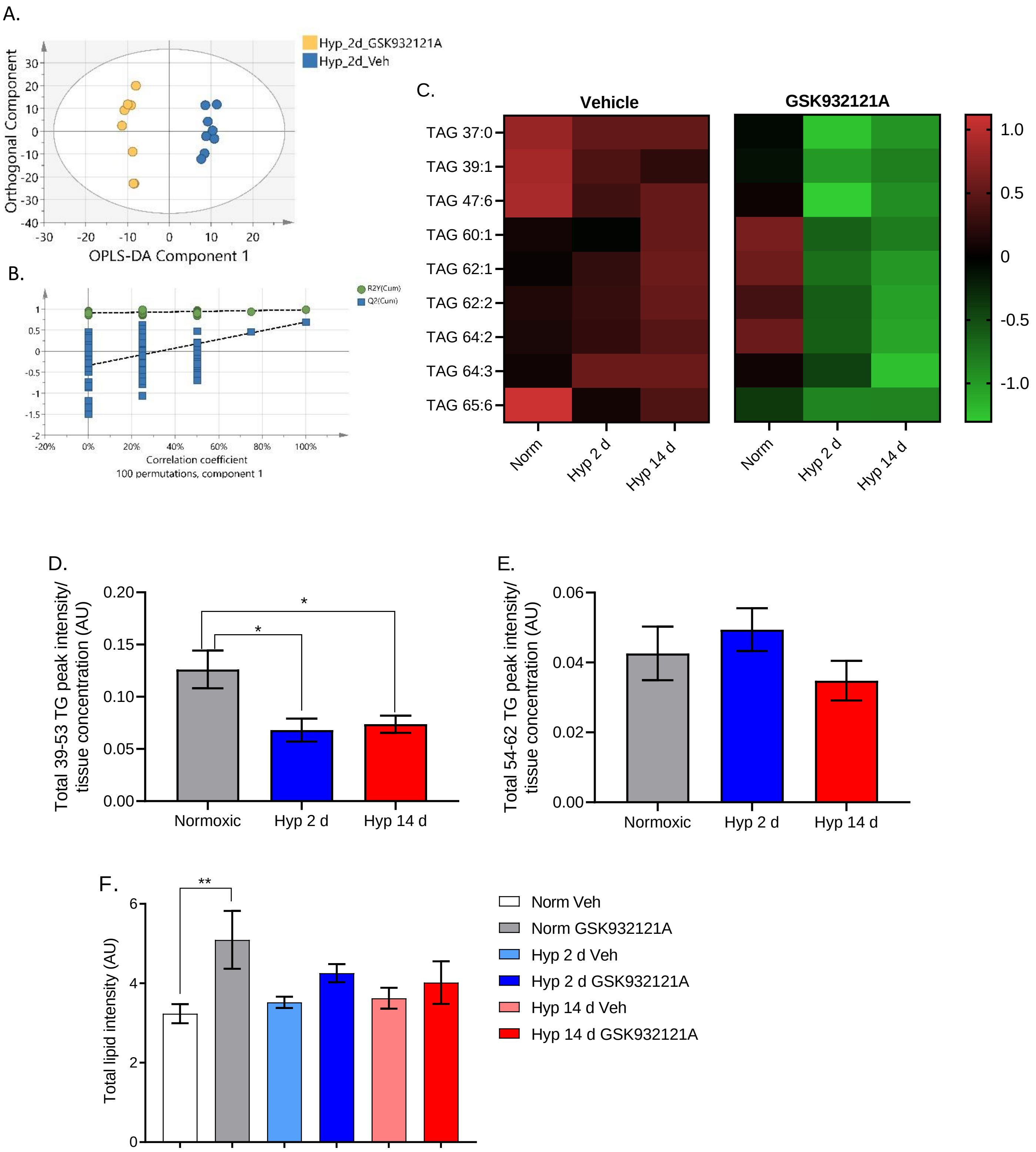

__Additional File 1: Fig S7.__ **Lipidomic profile of 2 d hypoxic Veh and GSK932121A treated animals, TAG levels with GSK932121A treatment and total lipid intensity.**

A. Orthogonal partial least squares – discriminant analysis (OPLS-DA) of hepatic lipidomic profiles from 2 day hypoxia Veh-treated (blue) and GSK932121A-treated (yellow) animals, with each dot representing the lipidomic profile of one animal. Data was obtained from the peak area ratio of LC-MS analysis, corrected to internal standards and protein concentration and normalised using Pareto scaling and generalised logarithm transformation. n = 6 per group.
B. Permutation validation of the plot in panel A.
C. Heatmap of TAG discriminants from OPLS-DA model in panel A. Discriminants defined as those lipids lying 2 standard deviations away from the mean on an S plot.
D. Total peak intensities of TAGs with chain lengths 39-53 in GSK932121A-treated normoxic, 2 d and 14 d treated animals. n = 7 for normoxic, n = 8 Hyp 2d, n = 6 Hyp 14 d.
E. Total peak intensities of TAGs with chain lengths 54-62 in GSK932121A-treated normoxic, 2 d and 14 d treated animals. . n = 7 for normoxic, n = 8 Hyp 2d, n = 6 Hyp 14 d.
F. Total lipid peak intensity obtained from positive and negative mode LC-MS data, including the following: fatty acids, glycerolipids (triacylglycerols, diacylglycerols), glycerophospholipids (phosphatidic acid, phosphatidylcholine, lysophosphatidylcholine, phosphatidylethanolamine, phosphatidylglycerol, phosphatidylinositol, phosphatidylserine), sphingolipid (sphingomyelin. ceramides) and sterols (cholesterol ester). Data are corrected to internal standard and tissue protein concentration, presented as arbitrary units (AU). n = 9 normoxic Veh, n = 7 normoxic GSK932121A-treated, n = 6 hypoxic 14 d GSK932121A-treated, n = 8 for remaining groups.

   Panels C-E, data is presented as mean ± SEM, * $p < 0.05$, ** $p \leq 0.01$.

Additional File 1: Fig S8.

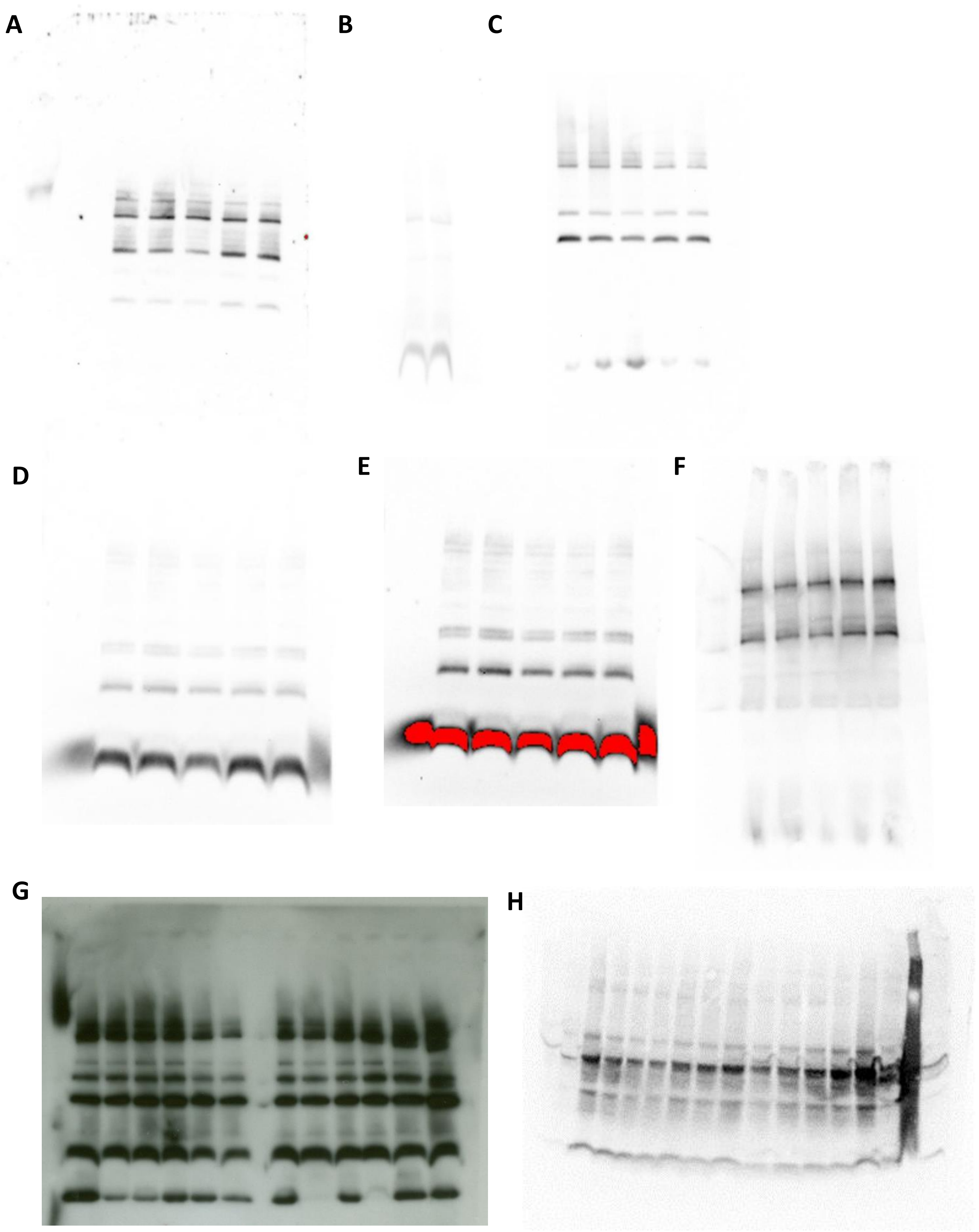

Additional File 1: Fig S8. Original immunoblot images

Original immunoblotting images for respiratory complexes I (A), II (B), III (C), IV (D and E), V (F) and all complexes using the OXPHOS cocktail (G) obtained using BN-PAGE, and Western Blotting (H). In the instance of complex IV, two exposures were required to clearly visualise upper and lower bands. These blots are presented in **Figure 2A**; (A), (C), (D), (E) and (F) are presented in **Additional File 1: Fig S3**; (H) is presented in **Additional File 1: Fig S4.**

Additional File 1: Fig S9.

A **B**

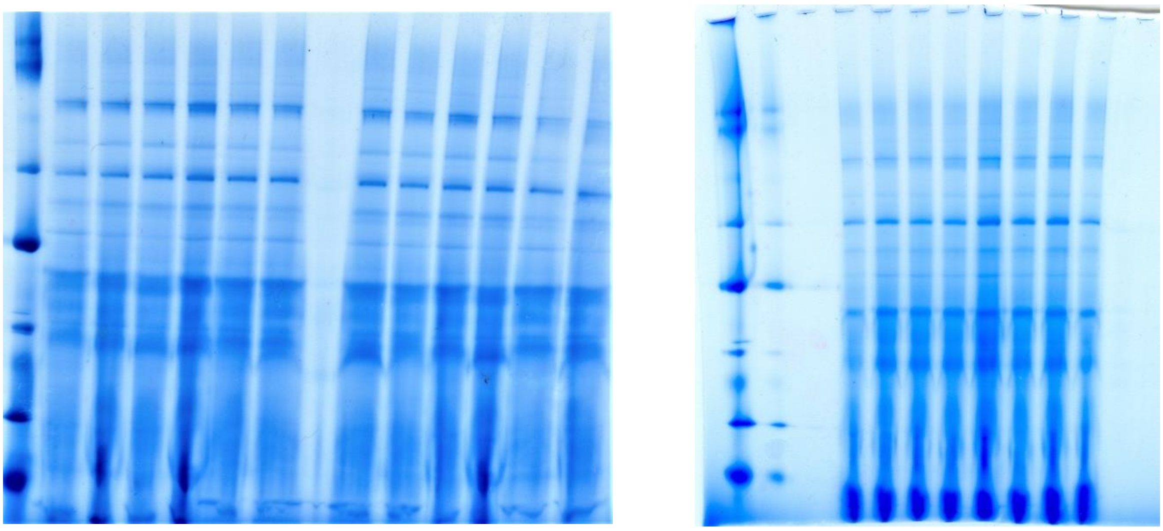

Additional File 1: Fig S9. Original colloidal blue stained images obtained using BN-PAGE

Original gel images from colloidal blue staining, obtained using BN-PAGE, as presented in **Figure 2A,B** (A) and **Additional File 1: Fig S3C** (B).
